# Systems biology informed neural networks (SBINN) predict response and novel combinations for PD-1 checkpoint blockade

**DOI:** 10.1038/s42003-021-02393-7

**Published:** 2021-07-15

**Authors:** Michelle Przedborski, Munisha Smalley, Saravanan Thiyagarajan, Aaron Goldman, Mohammad Kohandel

**Affiliations:** 1 Department of Applied Mathematics, University of Waterloo, Waterloo, ON Canada; 2 Integrative Immuno Oncology Center, Mitra Biotech, Woburn, MA USA; 3 Division of Engineering in Medicine, Brigham and Women’s Hospital, Boston, MA USA; 4 Department of Medicine, Harvard Medical School, Boston, MA USA

**Keywords:** Cancer immunotherapy, Systems biology, Machine learning

## Abstract

Anti-PD-1 immunotherapy has recently shown tremendous success for the treatment of several aggressive cancers. However, variability and unpredictability in treatment outcome have been observed, and are thought to be driven by patient-specific biology and interactions of the patient’s immune system with the tumor. Here we develop an integrative systems biology and machine learning approach, built around clinical data, to predict patient response to anti-PD-1 immunotherapy and to improve the response rate. Using this approach, we determine biomarkers of patient response and identify potential mechanisms of drug resistance. We develop systems biology informed neural networks (SBINN) to calculate patient-specific kinetic parameter values and to predict clinical outcome. We show how transfer learning can be leveraged with simulated clinical data to significantly improve the response prediction accuracy of the SBINN. Further, we identify novel drug combinations and optimize the treatment protocol for triple combination therapy consisting of IL-6 inhibition, recombinant IL-12, and anti-PD-1 immunotherapy in order to maximize patient response. We also find unexpected differences in protein expression levels between response phenotypes which complement recent clinical findings. Our approach has the potential to aid in the development of targeted experiments for patient drug screening as well as identify novel therapeutic targets.

## Abstract (web summary)

Michelle Przedborski et al. report an integrative systems biology and machine learning method for predicting cancer patient responses to immunotherapy treatment. Using their method, they identify several new drug combinations that could potentially improve treatment protocols for immunotherapy.

## Introduction

Recent advances in experimental techniques have enabled the collection of large clinical data sets to study the genomic and proteomic landscapes of an array of diseases and treatment strategies. Several computational approaches have been developed to analyze these high-dimensional data sets and elucidate biomarkers of disease presence, aggressiveness, or recurrence. In the context of cancer, systems biology approaches have been deployed to study protein and gene interaction networks^1–8^, to overcome drug resistance^9,10^, and to identify therapeutic targets^11–15^. On the other hand, genomic and proteomic data have been integrated into machine learning techniques for drug discovery and response prediction^16–18^ and for cancer risk prediction and cancer detection^19–24^. These approaches have individually shed light on a number of important questions in cancer biology. However, an integrative approach has the potential to provide a more complete picture, enabling researchers to simultaneously make inferences on novel drug targets, identify signatures of drug response, predict response to combination therapies, and determine optimal treatment protocols.

Anti-PD-1 immunotherapy is emerging as a targeted treatment strategy that has recently shown promise for several aggressive cancers, including melanoma, non-small-cell lung cancer (NSCLC), bladder, and head and neck cancers. This treatment strategy works by blocking the binding of PD-1 (programmed cell death protein 1) to PD-L1 (programmed death-ligand 1). Doing so prevents the inhibition of activated T lymphocytes (T-cells) and other immune cells^25^, which is caused by the over-expression of PD-L1 by the cancer cells and would otherwise allow the cancer cells to evade the immune response. Anti-PD-1 immunotherapy is the first immunotherapy to break through the historical 10% response rate ceiling associated with other immunotherapies, such as T-cell or dendritic cell vaccines^26^. Moreover, favorable patient response to immunotherapy tends to be durable and sustained for several years, meaning that immunotherapy has the potential to substantially improve patient prognosis. However, despite these promising findings, there is a high variability and unpredictability in the treatment outcome with anti-PD-1 immunotherapy, which is thought to be driven by patient-specific biology and the interactions of the patient’s immune system with the tumor.

We previously developed a systems biology approach to better understand how PD-1 blockade affects immune cell behavior and leads to different response dynamics^27^. The mathematical model was grounded on results from experiments of PD-1 blockade in a live ex-vivo human system, and the multi-disciplinary approach enabled the analysis and interpretation of the response dynamics of PD-1 blockade. However, this field of study is still missing an integrative computational approach for making discoveries in clinically relevant data sets that could help to identify biomarkers of response and to improve the patient response rate. An interdisciplinary approach, comprised of systems biology and machine learning techniques, is a natural choice of architecture for this endeavor for several reasons. First, experimentally calibrated systems biology models can generate an unlimited amount of meaningful simulated clinical data. Machine learning techniques require a large amount of training data to make accurate predictions that can be generalized to new data sets. Transfer learning^28^ can therefore be used to leverage small clinical data sets by pre-training on simulated data generated by the systems biology model. The systems biology model also enables the meaningful interpretation of the machine learning results, removing the so-called ‘black box’ dilemma^29^ associated with machine learning algorithms.

Here we leverage interdisciplinary science, integrating systems biology and machine learning approaches, to make a number of interesting discoveries that have the potential to substantially improve anti-PD-1 immunotherapy, including: empirical features that lead to response variability, mechanisms of drug resistance, novel drug combinations and optimal treatment protocols, and unexpected differences in protein expression between response phenotypes. Using systems biology informed neural networks (SBINN), we also calculate patient-specific parameter values and predict clinical outcome.

**Fig. 1:**
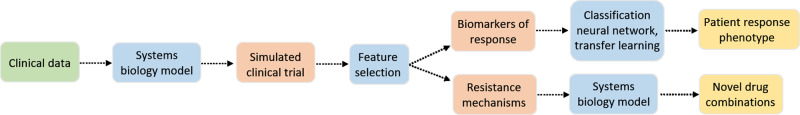
Overview of the data and methodology used throughout the work. A small clinical data set is used to develop and calibrate a systems biology model of anti-PD-1 immunotherapy, which is then employed to generate large simulated clinical trials. Feature extraction techniques are applied to the simulated data to determine biomarkers of response to therapy as well as potential mechanisms of drug resistance. The response features are used as inputs into a classification neural network (C-SBINN), which utilizes transfer learning, to predict the patient response phenotype. The identified mechanisms of intrinsic drug resistance are leveraged to develop novel drug combinations.

In brief, this is accomplished by using an experimentally calibrated systems biology model of anti-PD-1 immunotherapy to generate large simulated clinical trials. The simulated data is then analyzed using several feature extraction techniques to determine experimentally-measurable patient features that are important for distinguishing between response phenotypes, as well as potential mechanisms of drug resistance. The features are used as inputs into a classification neural network (C-SBINN), which is pre-trained on the simulated clinical trial data to classify virtual patients as responders or non-responders to anti-PD-1 immunotherapy. Next, we use transfer learning and apply the pre-trained neural network to real clinical data to predict the response phenotype of real patients. We also leverage the identified potential mechanisms of drug resistance to develop novel combination therapies to improve patient response to treatment. Our integrative approach, summarized in Fig. 1, is imperative for small clinical data sets, particularly, when the dimensionality of the experimental data exceeds the number of samples (patients). In this case, standard techniques cannot be used to extract important features of response to treatment, or to make predictions, directly from the clinical data.

The remainder of the paper is organized as follows. In the Results section, we give an overview of the main findings, then in the Discussion section we provide a detailed examination and suggest future research directions. Finally, in the Methods section, we give a detailed overview of the experimental and computational approaches that were used in the study.

## Results

### Feature selection identifies drug-induced changes in T-cell populations and cytokine expression as predictors of response phenotype

**Fig. 2:**
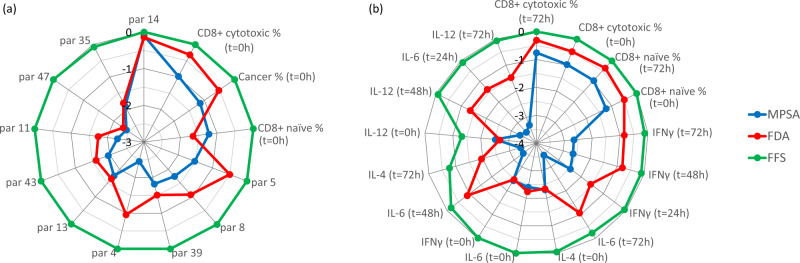
Distribution of feature selection scores from the MPSA method, Fisher discriminant analysis (FDA), and filter feature selection (FFS). **a** tumor micro-environment features and (**b**) experimentally accessible response features. Plots depict ${{\rm{log}}}_{10}[{\rm{MPSA}}\;{\rm{sensitivity}}]$, ${{\rm{log}}}_{10}[{\rm{standardized}}\;{\rm{canonical}}\;{{\rm{coefficient}}}]$ from FDA, and ${{\rm{log}}}_{10}$[1−*p*-value] obtained from two-sample *t*-test during FFS. In (**a**) systems biology kinetic parameter numbers are indicated by “par”.

We first investigated how features that characterize underlying patient-specific biology can be leveraged to predict clinical response to anti-PD-1 immunotherapy. To this end, we applied feature extraction techniques to the simulated clinical trial data, where the features were selected from the initial conditions and the systems biology kinetic parameters. This subset of features characterize the tumor micro-environment under control conditions. The resulting distributions of the top 10 tumor micro-environment feature selection scores for the MPSA method, Fisher discriminant analysis, and filter feature selection are depicted in Fig. 2a. In these radial plots, points that are furthest from the center contribute most to distinguishing between response and non-response phenotypes to anti-PD-1 immunotherapy. The figure illustrates the qualitative agreement between the three methods for the top-selected features.

The top 10 tumor micro-environment features predicted by the MPSA method and the ML feature selection techniques for distinguishing between response and non-response phenotypes to anti-PD-1 immunotherapy are also summarized in Table 1. Of all the features characterizing the underlying tumor micro-environment, the rate of killing of cancer cells by CD8+ cytotoxic T-cells (parameter 14) was determined to be the most significant kinetic parameter, and the initial CD8+ cytotoxic T-cell population the initial CD8+ naive T-cell population were determined to be the most significant experimentally measureable control features. Importantly, the ML results confirm the significant findings of the MPSA method. Moreover, as indicated in Table 1, several kinetic parameters related to IFN*γ* and IL-12 production were identified as significant using all three methods. Given that IFN*γ* and IL-12 both play a key role in CD4+ Th1 cell differentiation, see Supplementary Fig. 1, these results reinforce the experimental observation that the variability in patient response is connected to Th1-related changes^27^.

**Table 1:** Top 10 tumor micro-environment features selected by the MPSA method, Fisher discriminant analysis, and filter feature selection.

MPSA method	Fisher discriminant analysis	Filter feature selection
*Rate of cancer cell killing by cytotoxic* *CD8+ T-cells (14)*	*Rate of cancer cell killing by* *cytotoxic CD8+ T-cells (14)*	*Net proliferation rate of cancer* *cells (5)*
*Initial cytotoxic CD8+ T-cell* *level*	*Initial cytotoxic CD8+ T-cell* *level*	*Rate of cancer cell killing by* *cytotoxic CD8+ T-cells (14)*
*Initial cancer level*	*Net proliferation rate of cancer* *cells (5)*	*Initial cancer level*
*Initial naive CD8+ T-cell level*	*Initial cancer level*	*Initial naive CD8+ T-cell level*
*Net proliferation rate of cancer* *cells (5)*	IL-12-dependent net proliferation rate of cytotoxic CD8+T-cells (4)	*Initial cytotoxic CD8+ T-cell* *level*
*IL12-dependent growth rate of* *cytotoxic CD8+ T-cells (8)*	*IL-12-dependent growth rate of* *cytotoxic CD8+ T-cells (8)*	IL-12-dependent net proliferation rate of cytotoxic CD8+T-cells (4)
Rate of differentiation of naive CD8+ T-cells into cytotoxic CD8+ T-cells (13)	*Rate of dissociation of of PD-1:PD-L1 complex (39)*	*IL12-dependent growth rate of* *cytotoxic CD8+ T-cells (8)*
*Rate of dissociation of PD-1:PD-L1 complex (39)*	Half-maximal PD-1:PD-L1 concentration for inhibition of naive CD8+ T-cell differentiation into cytotoxic CD8+ T-cells (43)	Rate of IFN*γ* production by cytotoxic CD8+ T-cells (47)
Per-cell expression level of PD-1 (35)	*Initial naive CD8+ T-cell level*	*Rate of dissociation of PD-1:PD-L1 complex (39)*
Half-maximal PD-1:PD-L1 concentration for inhibition of naive CD8+ T-cell differentiation into cytotoxic CD8+ T-cells (43)	IL-12-dependent differentiation rate of naive helper CD4+ T-cells into type 1 helper T-cells (11)	IL-12-dependent differentiation rate of naive helper CD4+ T-cells into type 1 helper T-cells (11)

Features predicted by all three methods are italicized. The rate of killing of cancer cells by CD8+ cytotoxic T-cells was predicted to be the most significant kinetic parameter and the initial CD8+ cytotoxic T-cell population the initial CD8+ naive T-cell population were predicted to be the most significant experimentally-measureable features. Systems biology kinetic parameter numbers are indicated in parentheses, see Supplementary Table 2.

We next examined how changes in the tumor micro-environment due to anti-PD-1 immunotherapy correlate with the response phenotype. To this end, we again applied the feature selection techniques to the simulated clinical trial data, while also including the cytokine expression levels at *t* = 24, 48, 72 h during nivolumab treatment as well as the T-cell expression levels at *t* = 72 h. The feature selection scores for the experimentally measureable features that were determined to be most significant for distinguishing between response phenotypes using the MPSA method, Fisher discriminant analysis, and filter feature selection are presented in Fig. 2b. We further summarize the top 10 experimentally measureable features for each method in Table 2. Interestingly, the treatment-induced T-cell expression levels were predicted to be more important for stratifying response phenotype than the control levels or the kinetic parameters using Fisher discriminant analysis. Given that IL-4 and IL-6 has an inhibitory effect on Th1 cell production while IL-12 and IFN*γ* have a stimulatory effect, see Supplementary Fig. 1, the results in Table 2 suggest that the opposing interactions are most significant for stratifying response dynamics. Furthermore, these results reinforce the previous experimental observation implicating Th1-related changes in anti-PD-1 immunotherapy response^27^. Furthermore, IL-12 plays a key role in cytotoxic CD8+ T-cell proliferation, see Supplementary Fig. 1, which is evidently a strong contributor to the response phenotype.

**Table 2:** Top 10 experimentally-accessible response features selected by the MPSA method, Fisher discriminant analysis, and filter feature selection for distinguishing responder and non-responder phenotypes to anti-PD-1 immunotherapy.

MPSA method	Fisher discriminant analysis	Filter feature selection
*Cytotoxic CD8+ T-cell level* *at* *t* = 72 *h*	*Cytotoxic CD8+ T-cell level* *at* *t* = 72 *h*	*Initial naive CD8+ T-cell* *level*
*Initial cytotoxic CD8+ T-cell* *level*	*Naive CD8+ T-cell level at* *t* *=* *72* *h*	*Initial cytotoxic CD8+ T-cell* *level*
*Naive CD8+ T-cell level at* *t* *=* *72* *h*	*Initial cytotoxic CD8+ T-cell* *level*	*Naive CD8+ T-cell level at* *t* *=* *72* *h*
*Initial naive CD8+ T-cell level*	*Initial naive CD8+ T-cell level*	*Cytotoxic CD8+ T-cell level* *at t* *=* *72* *h*
Initial IL-4 expression	*IFNγ* *expression at* *t* = 72 *h*	*IFNγ* *expression at* *t* = 72 *h*
Initial IL-6 expression	*IFNγ* *expression at* *t* = 48 *h*	*IFNγ* *expression at* *t* = 48 *h*
Initial IFN*γ* expression	IL-6 expression at *t* = 48 h	*IFNγ* *expression at* *t* = 24 *h*
*IFNγ* *expression at* *t* = 24 *h*	*IL-6 expression at* *t* = 72 *h*	IL-4 expression at *t* = 72 h
Initial IL-12 expression	IL-12 expression at *t* = 48 h	*IL-6 expression at* *t* = 72 *h*
*IFNγ* *expression at* *t* = 48 *h*	IL-6 expression at *t* = 24 h	IL-12 expression at *t* = 72 h

Features that were predicted by at least two of the methods are italicized.

### Regression neural network accurately calculates the most significant kinetic parameter

As described above, the rate of cancer cell killing by cytotoxic CD8+ T-cells *k*_*c*_ (parameter 14) was identified to be the most significant kinetic parameter for discriminating between response phenotypes. Sampling the values of *k*_*c*_ logarithmically during the generation of the simulated clinical trial led to Pearson correlation coefficients between parameter 14 and IFN*γ* expression levels at *t* = 0, 24, 48, and 72 h with values of, respectively, 0.609, 0.0560, 0.0131, and 0.0071. All other Pearson correlation coefficients were <0.0030 in magnitude. Using the IFN*γ* expression levels as inputs, the non-linear regression systems biology informed neural network (NR-SBINN) calculated the logarithm of *k*_*c*_ with an error of 0.06 ± 0.48% on the training data and 0.06 ± 0.22% on the testing data. The corresponding learning curve for the NR-SBINN is illustrated in Supplementary Fig. 3 and depicts the loss function quickly falling to near zero on both the training and validation data sets. The predicted patient-specific *k*_*c*_ values, obtained by applying the trained NR-SBINN to the ex-vivo data set, had an average value of 9.3 × 10^−8^, standard deviation of 3.6 × 10^−7^, and fell within the range of 1.0 × 10^−8^ to 2.2 × 10^−6^ T_c_ cell^−1^ ⋅ day^−1^.

### Transfer learning improves the response phenotype prediction accuracy

The most significant tumor micro-environment features (the patient-specific *k*_*c*_ value, the initial CD8+ cytotoxic T-cell population, and the initial CD8+ naive T-cell population) were used as inputs into the classification systems biology informed neural network (C-SBINN) to predict response phenotype to anti-PD-1 immunotherapy directly from these features. The response phenotype prediction results are presented in Table 3 for the imbalanced clinical trial data, for the direct application of the pre-trained C-SBINN to the ex-vivo data (i.e. before the transfer learning step), and for the ex-vivo data after the transfer learning. For comparison, we also present the classification results for the ex-vivo data without transfer learning. As described in the Machine learning protocol: classification neural network section, the optimal neural network architecture and learning parameters leading to these results are distinct for the two cases with and without transfer learning (see also Supplementary Section E).

**Table 3:** Classification results obtained by using the tumor micro-environment features as inputs to the C-SBINN.

*I. Imbalanced simulated clinical trial data*						
ROC (AUC)	PRC (AUC)	MCC	CKS	MCC (all)	CKS (all)	Optimal threshold
0.90	0.80	0.72	0.68	0.68	0.63	0.22
*II. Direct application to ex-vivo patient data before transfer learning step*						
ROC (AUC)	PRC (AUC)	MCC	CKS	MCC (all)	CKS (all)	Optimal threshold
0.70	0.34	–	–	0.37	0.35	0.25
*III. Ex-vivo patient data, with transfer learning*						
ROC (AUC)	PRC (AUC)	MCC	CKS	MCC (all)	CKS (all)	Optimal threshold
0.84 ± 0.13	0.59 ± 0.28	0.74 ± 0.16	0.69 ± 0.19	0.49 ± 0.11	0.48 ± 0.11	0.14 ± 0.09
*Ex-vivo patient data, without transfer learning*						
ROC (AUC)	PRC (AUC)	MCC	CKS	MCC (all)	CKS (all)	Optimal threshold
0.61 ± 0.28	0.45 ± 0.33	0.49 ± 0.33	0.44 ± 0.35	0.11 ± 0.14	0.10 ± 0.11	0.18 ± 0.12

ROC (AUC) is the area under the receiving-operator characteristic curve, PRC (AUC) is the area under the precision-recall curve, CKS is the Cohen kappa score, and MCC is the Matthew correlation coefficient (see the Machine learning protocol: performance metrics section). Average and standard deviation obtained from 10-fold cross validation are shown. MCC (all) and CKS (all) refer to an additional validation step wherein the trained C-SBINN was applied to the entire data set after optimizing the classification threshold on the testing set only. For comparison, we show the results of direct application of the pre-trained network to all ex-vivo data before the transfer learning step.

**Fig. 3:**
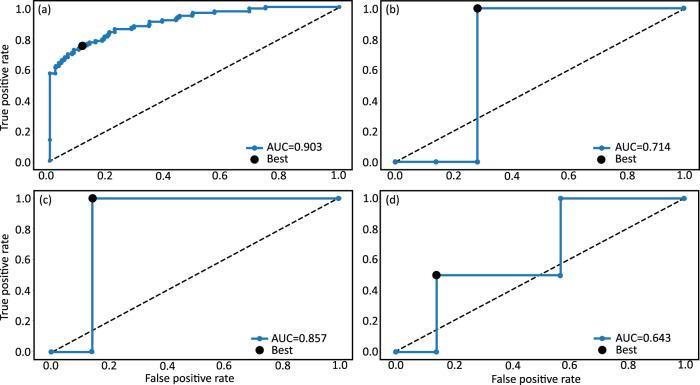
Representative ROC curves for response phenotype classification. **a** imbalanced simulated clinical data set, **b** application of pre-trained C-SBINN directly to ex-vivo data before the transfer learning step, **c** ex-vivo data set with transfer learning, and **d** ex-vivo data set without transfer learning. Displayed curves were selected from the 10-fold cross validation to approximately coincide with the mean AUC, see Table 3. Best g-mean score is highlighted on all plots.

We see from Table 3 that, based on the ROC (AUC) results, the C-SBINN has excellent prediction accuracy on the simulated clinical trial data and ex-vivo data with transfer learning. Indeed, the CKS values in both cases fall within the range of substantial agreement^30^ between the C-SBINN phenotype prediction and the true response phenotype. Furthermore, it is evident from Table 3 that the transfer learning approach, i.e. pre-training the C-SBINN on the simulated data, results in a significant increase in the prediction accuracy on the ex-vivo data set in comparison to without transfer learning: there was on average a 38% increase in ROC (AUC), 31% increase in PRC (AUC), a 51% increase in MCC, and a 57% increase in CKS on the testing set. To quantify the significance of the improvement, we performed a one-sided non-parametric Mann–Whitney *U* Test on the individual scores obtained with and without transfer learning. The test results indicated that the differences obtained from transfer learning are statistically significant, with the following *p*-values: ROC (AUC) *p* < 0.035, PRC (AUC) *p* < 0.075, MCC *p* < 0.043, CKS *p* < 0.043, MCC (all) *p* < 0.00013, CKS (all) *p* < 0.00017. Representative ROC curves, selected to reflect the average area under the curve displayed in Table 3, are depicted in Fig. 3.

Since the patient-specific *k*_*c*_ values were calculated from the IFN*γ* expression at four time points, taking the three most significant tumor micro-environment features as inputs into the C-SBINN required using a total of six experimental measurements for each patient. While this is a small subset of features that may give rise to the possibility of more targeted ex-vivo screening experiments, one drawback to this approach is that it necessitates the calculation of a patient-specific kinetic parameter. To overcome this limitation, we instead utilized the top six experimentally measurable response features as inputs to the C-SBINN to predict the response phenotype for each patient. The results with and without transfer learning are, respectively, tabulated in the first two rows of Table 4. Surprisingly, not including the top kinetic parameter as an input feature in the C-SBINN resulted in a lower prediction accuracy for the simulated data. However, the C-SBINN still obtained a good prediction accuracy on the ex-vivo data with transfer learning, on average performing similarly to the case of the tumor micro-environment features, c.f. Tables 3 and 4. Furthermore, as indicated in Table 4, the transfer learning step results in on average a 9% increase in ROC (AUC), a a 19% increase in PRC (AUC), a 16% increase in MCC, and a 21% increase in CKS on the testing set, compared to without transfer learning. To quantify the significance of the difference between approaches, we again performed a one-sided non-parametric Mann–Whitney *U* test on the individual scores obtained with and without transfer learning. The test results indicated that the differences obtained from transfer learning are not as statistically significant as with the tumor micro-environment features. In particular, the following *p*-values were obtained: ROC (AUC) *p* < 0.12, PRC (AUC) *p* < 0.15, MCC *p* < 0.12, CKS *p* < 0.11, MCC (all) *p* < 0.83, CKS (all) *p* < 0.75.

**Table 4:** Ex-vivo classification results from 10-fold cross validation using the top six experimentally accessible response features as inputs to the C-SBINN.

*Top 6 features selected from simulated data, with transfer learning*						
ROC (AUC)	PRC (AUC)	MCC	CKS	MCC (all)	CKS (all)	Optimal threshold
0.82 ± 0.16	0.62 ± 0.28	0.72 ± 0.21	0.68 ± 0.22	0.63 ± 0.12	0.61 ± 0.13	0.28 ± 0.12
*Top 6 features selected from simulated data, without transfer learning*						
ROC (AUC)	PRC (AUC)	MCC	CKS	MCC (all)	CKS (all)	Optimal threshold
0.75 ± 0.19	0.52 ± 0.27	0.62 ± 0.22	0.56 ± 0.25	0.63 ± 0.20	0.59 ± 0.23	0.31 ± 0.28
*Top 6 features selected from ex-vivo data, without transfer learning*						
ROC (AUC)	PRC (AUC)	MCC	CKS	MCC (all)	CKS (all)	Optimal threshold
0.71 ± 0.20	0.59 ± 0.27	0.59 ± 0.25	0.55 ± 0.28	0.39 ± 0.16	0.36 ± 0.16	0.78 ± 0.33
*All ex-vivo experimental measurements, without transfer learning*						
ROC (AUC)	PRC (AUC)	MCC	CKS	MCC (all)	CKS (all)	Optimal threshold
0.75 ± 0.13	0.39 ± 0.20	0.59 ± 0.17	0.52 ± 0.21	0.64 ± 0.13	0.60 ± 0.17	0.25 ± 0.23

Average and standard deviation are shown. First two rows correspond to response features selected from the simulated clinical trial data. Third and fourth rows correspond to, respectively, the top six experimental features selected directly from the ex-vivo data and all the experimental ex-vivo measurements. MCC (all) and CKS (all) refer to an additional validation step wherein the trained C-SBINN was applied to the entire ex-vivo data set after optimizing the classification threshold on the testing set only.

We were next interested in determining whether the features selected by the systems biology model are indeed the optimal features for stratifying response phenotypes. To this end, we investigated the response phenotype prediction accuracy using the top six experimental features that were selected directly by the ex-vivo patient data set at each fold of cross-validation. Over all cross-validation folds, the most common features that were selected by Fisher discriminant analysis are: the IFN*γ* expression at *t* = 48 h, IFN*γ* expression at *t* = 24 h, initial naive CD8+ T-cell level, IFN*γ* expression at *t* = 72 h, IL-6 expression at *t* = 24 h, the naive CD8+ T-cell level at *t* = 72 h, the cytotoxic CD8+ T-cell level at *t* = 72 h, and the initial IFN*γ* expression. The results are shown in Table 4 and indicate that the features selected by the ex-vivo data set lead to a similar prediction accuracy as the response features selected by the systems biology model without transfer learning. Specifically, the features selected by the ex-vivo data set led to a higher score for some performance metrics, such as the PRC (AUC), while leading to lower scores for other metrics, such as the ROC (AUC), MCC, and CKS on the testing sets and the final validation step. Finally, in Table 4 we present the classification results obtained by using all of the experimental measurements in the ex-vivo data set as inputs into the neural network. Comparison of these results with the other cases in Table 4 indicates that including all of the experimental features does not improve the performance on the testing set, which is likely due to an increase in noise variables that are not helpful for stratifying the response phenotypes. Importantly, we point out that the optimal network architecture and learning parameters are distinct for each case considered in Table 4 (see Machine learning protocol: classification neural network section and Supplementary Section E).

### Optimal timing of combined IL-6 inhibition and recombinant IL-12 considerably improve patient response to anti-PD-1 immunotherapy

As illustrated above, coupling systems biology with machine learning enables a deeper understanding of the underlying biological system of interest, and is particularly advantageous when the experimental data set is small. The feature selection results indicated that drug-induced changes in the naive and cytotoxic CD8+ T-cell levels are important for distinguishing response phenotypes to anti-PD-1 immunotherapy. Temporally evolving competitive cytokine interactions were also found to play a significant role, where IL-4 and IL-6 positively regulate CD4+ Th2 cell production and negatively regulate CD4+ Th1 cell production, while IL-12 and IFN*γ* have a stimulatory effect on the production of CD4+ Th1 cells and cytotoxic CD8+ T-cells, see Supplementary Fig. 1. Building on these insights, here we investigate mechanisms for shifting the competing cytokine interactions in favor of CD4+ Th1 cell and cytotoxic CD8+ T-cell production to improve patient response to anti-PD-1 immunotherapy. Given that IL-6 inhibitors and recombinant IL-12 have individually shown promise for improving the anti-tumor effects of, respectively, PD-1 and PD-L1 blockade, see for example refs. ^31,32^, we used the systems biology model to examine the timing of their individual and combined immunomodulatory effects, to ultimately determine the optimal treatment protocol leading to maximal patient response.

**Fig. 4:**
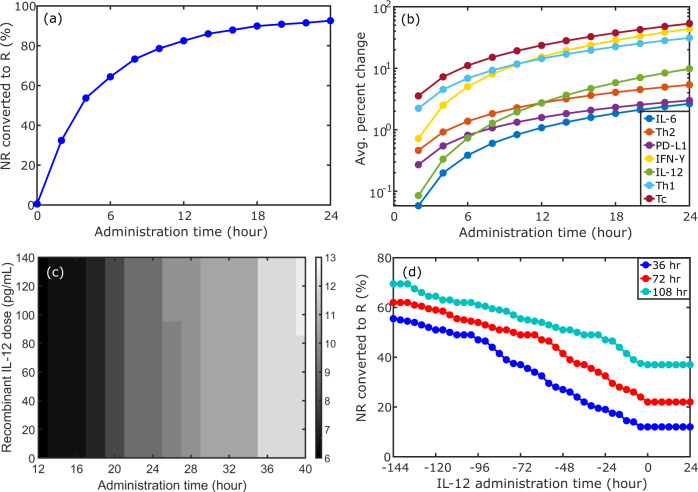
Simulation results of IL-6 inhibitor and recombinant IL-12 combined with the nivolumab treatment protocol for non-responders to anti-PD1 immunotherapy. **a** Percent of patients converted from non-responders to responders at a fixed single dose of 10 pg/mL recombinant IL-12 administered at different times *before* nivolumab treatment protocol. **b** Corresponding absolute value of average percent change of cytokine and T-cell populations. Average was taken over 1000 patients, calculated between the time of recombinant IL-12 administration and just prior to nivolumab treatment protocol. For IL-6, Th-2, and PD-L1, average percent change is negative, corresponding to inhibition; for all other quantities, average percent change is positive. Change in the IL-12 level does not include the recombinant IL-12 concentration. For (**a**, **b**), the 1000 patients were chosen from the non-responder group by selecting patients with the smallest tumor size after the nivolumab treatment protocol (note that the tumor size was larger than before nivolumab treatment per our definition of “non-responder”). **c** Percent of 200 randomly selected non-responders converted to responders as a function of single dose strength of recombinant IL-12 and the administration time. Minimum dose administered was 1 pg/mL. For (**a**–**c**), administration time is the number of hours recombinant IL-12 was given prior to the start of the nivolumab treatment protocol. For IL-6 inhibition, corresponding plots (**a**–**c**) are very similar, see Supplementary Fig. 2. **d** Triple combination therapy comprised of single dose 10 pg/mL recombinant IL-12, single dose IL-6 inhibitor (maximum 1% reduction in IL-6 production rate), and nivolumab treatment protocol. Percent of 200 randomly selected non-responders converted to responders as a function of recombinant IL-12 administration time for different IL-6 inhibition times is shown. IL-6 inhibitor was given before nivolumab treatment, and the reported legend times are the hours administered prior to the start of nivolumab treatment protocol. IL-12 administration times are measured relative to the particular IL-6 administration time.

In Fig. 4a, we show the percentage of 1000 non-responders to anti-PD-1 immunotherapy that were converted to responders by administering a single dose of 10 pg/mL recombinant IL-12 at different times before the start of nivolumab treatment. As Fig. 4a indicates, administering recombinant IL-12 concurrently with nivolumab treatment (administration time = 0 h), does not lead to any patients being converted to responders. On the other hand, administering recombinant IL-12 1-day before the start of nivolumab treatment (administration time = 24 h) leads to almost all of the 1000 non-responders being converted to responders to anti-PD-1 immunotherapy. The importance of the temporal delay is reinforced by Fig. 4b, which indicates that it takes at least 24 h for the recombinant IL-12 to substantially alter the tumor micro-environment. At this point, there is a slight decrease in IL-6 and PD-L1 levels and a modest decrease in CD4+ Th2 cell levels. In comparison, there is a modest increase in IL-12 production, and a substantial increase in IFN*γ*, CD4+ Th1 cells, and cytotoxic CD8+ T-cell levels, which makes the tumor micro-environment more favorable for responding to anti-PD-1 immunotherapy. An identical timing study was conducted for IL-6 inhibition, where the reduction in IL-6 production rate was taken to be 1% at maximal IL-6 inhibitor concentration, and the results were quantitatively very similar, see Supplementary Fig. 2, indicating that the effects are maximized when the inhibitor is administered well before the start of immunotherapy.

Next we examined how changing both the administration time and the concentration of single dose recombinant IL-12 affects the conversion of non-responders to responders to anti-PD-1 immunotherapy. The results are presented in Fig. 4c for 200 randomly selected non-responders and indicate that the conversion to response phenotype is more sensitive to the administration time than the dose strength, increasing from 6% converted at 12 h prior to nivolumab administration to 13% converted at 40 h prior to nivolumab administration. However, there is a slight sensitivity to the dose strength at the 26 and 40 h administration times, see Fig. 4c. The results were quantitatively similar for an identical study involving IL-6 inhibition, in which the IL-6 inhibitor was administered at different times before nivolumab treatment, and different dose strengths were assumed to reduce the IL-6 production rate between 1% and 60% at maximal IL-6 inhibitor concentrations. Importantly, these findings reinforce the main results in Fig. 4a, b, specifically, that it is crucial to administer the recombinant IL-12 or the IL-6 inhibitor well in advance of the start of immunotherapy to maximize their effects.

Building on these results, we further considered the optimization of triple combination therapy, in which a fixed single dose of recombinant IL-12 and a fixed single dose of IL-6 inhibitor were given separately prior to the start of nivolumab treatment. For this study, 200 non-responders were randomly selected and the fraction of non-responders that were converted to responders for different IL-12 and IL-6 administration times was examined. The results are presented in Fig. 4d and indicate that when IL-12 is administered after the IL-6 inhibitor, the fraction of non-responders that are converted to responders saturates at a minimum constant value that depends on the IL-6 administration time. In this case, the combination of IL-6 and IL-12 is no more effective than administering either individual therapy alone prior to the nivolumab treatment protocol, c.f. Fig. 4c. However, by increasing the IL-6 inhibitor administration time, this minimum constant value increases considerably, achieving close to 40% of non-responders when IL-6 is inhibited 108 hours prior to nivolumab treatment and recombinant IL-12 is administered at the same time or after IL-6 inhibition. These results support the finding that it is necessary to administer the recombinant IL-12 and an IL-6 inhibitor well in advance of the start of anti-PD-1 immunotherapy so that there is sufficient time for the tumor micro-environment to be reconfigured into a manner which is more conducive to treatment response.

Importantly, we see from Fig. 4d that administering recombinant IL-12 prior to IL-6 inhibition is the most efficient treatment protocol, leading to ~70% of randomly selected non-responders being converted to responders to anti-PD-1 immunotherapy when the IL-6 inhibitor is administered 108 h before nivolumab treatment and recombinant IL-12 is administered 144 h prior to IL-6 inhibition. Finally, it is interesting to note that if the curves in Fig. 4 are plotted as a function of the IL-12 administration time, measured instead with respect to the start of the nivolumab treatment protocol, then the curves overlap to the left of where they deviate from their respective minimum constant values. This is an interesting finding because it indicates that an identical response can be achieved for shorter IL-6 inhibitor administration times by sufficiently increasing the recombinant IL-12 administration time. This supports the notion that the micro-environmental changes resulting from recombinant IL-12 administration are long lasting, though further investigation would be required to confirm this hypothesis.

### Predicted response phenotype correlates with high Ki-67 expression

**Fig. 5:**
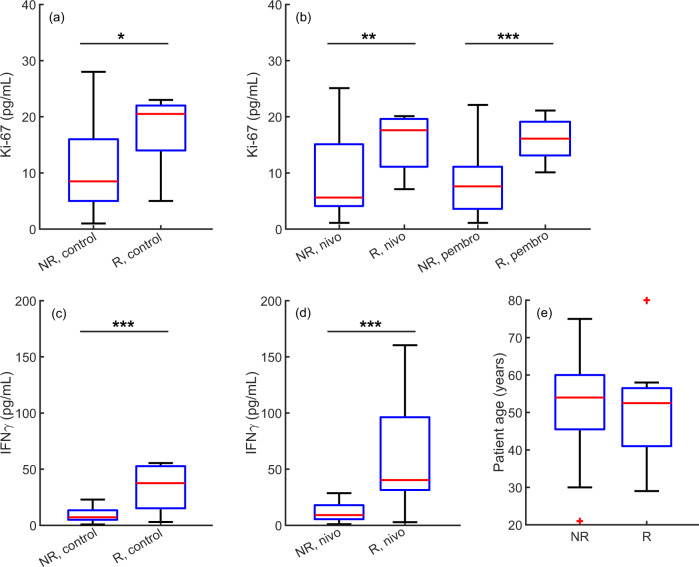
Statistically significant differences between patients classified as “responders” (R) and “non-responders” (NR) under vehicle control, nivolumab (nivo) treatment, and pembrolizumab (pembro) treatment conditions. **a**, **b** Average ex-vivo Ki-67 expression. **c**, **d** Average ex-vivo IFN*γ* expression. ^*^p < 0.02, ^**^p < 0.05, ^***^p < 0.01. In **a**–**d**, outliers were removed for visual clarity. There were no statistically significant age differences between the responder and non-responder groups (**e**).

Lastly, we attempted to learn more about how patient-specific biology correlates with response phenotype, beyond the features that were captured by the systems biology model. To this end, we performed a statistical analysis of a comprehensive experimental ex-vivo data set, in search of previously undetected statistically significant differences between the patients who were predicted to be ‘responders’ to anti-PD1 immunotherapy and those predicted to be ‘non-responders’. Fisher’s exact test indicated that there was no nonrandom association between patient sex and response phenotype, i.e. male and female patients do not have different odds of responding to anti-PD-1 immunotherapy. Similarly, patient age was not found to be a determining factor in the response to anti-PD-1 immunotherapy using Fisher’s exact test or two-sample Kolmogorov–Smirnov (KS) and Mann–Whitney *U* (MWU) tests, see Fig 5. However, two-sample KS and MWU tests both indicated that, at the 5% significance level, there were statistically significant differences in the distribution of the the tumor control, nivolumab-treated, and pembrolizumab-treated average Ki-67 isotype expression (*p*-values: 0.0109, 0.0346, 0.0015 (KS); 0.0147, 0.0233, 0.0018 (MWU), respectively) and the control and nivolumab-treated average IFN*γ* expression (*p*-values: 0.0080, 0.0015 (KS); 0.0102, 0.0041 (MWU), respectively), see Fig. 5. In addition, the granzyme and perforin levels were significantly higher in the predicted ‘responder’ phenotype (*p*-values: 0.0346, 0.0030 (KS); 0.0107, 0.0009 (MWU), and 0.0263, 0.0109 (KS); 0.0091, 0.0036 (MWU), respectively), see Supplementary Fig. 5.

The granzyme and perforin results were expected, given that granzyme and perforin expression were used to stratify the patients into different response groups, see Supplementary Section F. In comparison, the Ki-67 results were surprising, since the expression was significantly higher in the predicted responder phenotype under all treatment conditions, see Fig. 5a, b. This result is interesting because Ki-67 is a biomarker for cellular proliferation. Therefore, under control conditions, high Ki-67 expression could indicate a highly proliferative, more aggressive cancer. In contrast, anti-PD-1 immunotherapy has previously been shown to induce the proliferation of tumor-specific CD8+ T-cells^33–35^, which leads to increased expression of Ki-67 in immune cells in the tumor micro-environment and peripheral blood. Indeed, recent clinical findings for patients with NSCLC treated with anti-PD-1 immunotherapy found that a higher Ki-67 expression level among PD-1+ CD8+ T-cells was a predictor for superior durable clinical benefit and survival rate^35,36^.

## Discussion

Interdisciplinary approaches are crucial for analyzing and interpreting the high-dimensional data sets that arise in experimental studies of biological systems. Machine learning techniques are particularly well-suited for these tasks, whether it be for classification, clustering, dimensionality-reduction, or for the identification of features that contribute significantly to variations between different classes or response phenotypes. However, while machine learning approaches are extraordinarily versatile, they require a large amount of training data to be highly accurate and generalizable to new data sets. This can present a considerable challenge if financial or experimental limitations prohibit the generation of sufficiently large biological or clinical data sets to be used for both training and testing purposes. Fortunately, as shown in this work, experimentally-calibrated systems biology models can remedy this shortcoming by generating biologically relevant simulated clinical trial data sets. Beyond this, systems biology approaches provide tools that are instrumental for aiding in the understanding of complex biological systems, with applications ranging from, for example, identifying novel drug targets to optimizing combination treatment protocols.

Indeed, in this work we illustrated several methods for how systems biology can be integrated with machine learning approaches to improve patient response to anti-PD-1 immunotherapy. Using our experimentally calibrated systems biology model, we first generated several large simulated clinical trial data sets. We then showed, using feature selection techniques, that the model captures the variability in patient response dynamics to anti-PD-1 immunotherapy and also selects features that confirm previous experimental findings^27^. Importantly, the results indicated that, in addition to the identified key model kinetic parameters, there are several experimentally measureable patient control features that are important for distinguishing the response phenotype, including the levels of cytotoxic and naive CD8+ T-cells. This is a notable finding because the patient control features reflect the underlying tumor micro-environment. Therefore, these results give insight into how to efficiently characterize inter-patient heterogeneity in the tumor micro-environment. Moreover, the experimental accessibility of these features means that they can be directly probed to identify potential mechanisms of intrinsic or adaptive drug resistance^37,38^ prior to the administration of therapy. This approach may therefore provide insights into novel therapeutic targets that can potentially help alter the tumor micro-environment into a setting which is more favorable for treatment response.

Building on the results, we developed a transfer learning approach, C-SBINN, to predict patient response using the features that were identified as most significant for distinguishing response phenotypes. The C-SBINN approach relied on pre-training the neural network with simulated clinical trial data that was generated using the experimentally-calibrated systems biology model. This approach significantly improved the ability of the neural network to predict ex-vivo patient response phenotype, compared to training on the experimental data alone. This is because when the ex-vivo data set is small, the patient samples are not reflective of all the possible biologically relevant parameter combinations and initial conditions that characterize the heterogeneity of the tumor micro-environment. Thus, by pre-training the C-SBINN on simulated clinical trial data that exhibits a wider range of initial conditions and combinations of parameter values, the network is better able to generalize to new data points (see also Supplementary Section E).

Using the C-SBINN, we first aimed to predict patient response phenotype from the subset of features that characterizes the tumor micro-environment. This approach can help to reduce the complexity and cost of experiments, potentially giving rise to more targeted ex-vivo patient drug screening. However, optimizing the prediction accuracy of the C-SBINN in this case required the top kinetic parameter, *k*_*c*_, to be used as an input feature into the network. Consequently, we developed a non-linear regression neural network approach, the NR-SBINN, to calculate the patient-specific values of *k*_*c*_. The NR-SBINN was trained on a simulated clinical trial data set that was generated using the systems biology model, wherein only the parameter value and the initial conditions were varied, and the strongest correlations were artificially inflated during the sampling process. This sampling may result in stronger correlations than those exhibited within the clinical data; however, it significantly improved the prediction accuracy of the NR-SBINN and reduced the amount of experimental features that were required for the calculation. While there are other methods available, such as the genetic algorithm, to estimate patient-specific kinetic parameter values, these procedures must be repeated for each new experimental sample. In contrast, the trained NR-SBINN can generalize to new patient data without the need for additional optimization or training. However, more analysis is required to investigate whether the NR-SBINN approach is identifiable, and how its identifiability is related to the systems biology model. One solution for detecting and alleviating potential issues with practical non-identifiability would be to incorporate the set of ODEs comprising the systems biology model directly into the NR-SBINN loss function^39^.

We also predicted the patient response phenotype using the C-SBINN transfer learning approach with the the top experimentally measureable response features as inputs. This approach required experimental determination of the treatment-induced changes in the cytotoxic and naive CD8+ T-cell populations and it resulted in an improved prediction accuracy on the ex-vivo data set. Importantly, despite not achieving as high of a prediction accuracy on the simulated clinical data in this case, the C-SBINN still benefited from exposure to the wide array of tumor micro-environments captured by the sampled combinations of parameter values and initial conditions. Moreover, the response prediction accuracy was found to be at least as high as when the classification network input features were selected directly by the ex-vivo data. This is a substantial finding because if the number of patient samples is small compared to the number of experimentally measured features, feature selection techniques can not be applied directly to the experimental data set. In this case, it is important to have another approach that can determine which experimentally measured features most distinguish the response phenotypes.

We have seen that coupling systems biology with machine learning approaches enables a deeper understanding of the biological system, and this integrative technique is particularly advantageous when the experimental data set is small. One major benefit to this approach is that it removes the so-called “black box” effect associated with machine learning algorithms, since the experimentally calibrated systems biology model can be used to provide context to machine learning results. For example, the machine learning feature selection techniques identified the control cytotoxic and naive CD8+ T-cell populations as being two of the top three tumor micro-environment features that distinguish response phenotypes. Informed by the systems biology model, we see from Supplementary Fig. 1, that there are several mechanisms that can lead to insufficient cytotoxic or naive CD8+ T-cell populations, driving intrinsic resistance to anti-PD-1 immunotherapy. These mechanisms of intrinsic drug resistance can be further exploited to improve patient response to anti-PD-1 immunotherapy. For example, inspection of the molecular interactions in the systems biology model indicates that inhibiting IL-6 would effectively reduce the CD4+ Th2 cell population and increase the CD4+ Th1 population by enabling the positive feedback from IFN*γ*, ultimately leading to an increased CD8+ cytotoxic T-cell population. Similarly, directly increasing IL-12 expression would lead to a higher CD4+ Th1 cell population and a higher cytotoxic CD8+ T-cell population. These insights provided a rationale for the development of a novel triple combination therapy to improve patient response to anti-PD-1 immunotherapy.

In previous studies^31,32^, IL-6 inhibitors and recombinant IL-12 individually showed promise for improving the anti-tumor effects of, respectively, PD-1 and PD-L1 blockade. Given these results and insights from the systems biology model, we sought to investigate the ideal treatment protocol for triple combination therapy consisting of IL-6 inhibition, recombinant IL-12, and anti-PD-1 immunotherapy. Interestingly, the efficiency of the treatment protocol showed only weak sensitivity to the strength of the IL-6 and IL-12 drug doses. Further experimental studies would be required to determine whether this behavior is strictly due to the choice of Hill functions to model the stimulatory and inhibitory immune interactions in the systems biology model, see Supplementary Sections A and C. In comparison, the efficiency of the triple combination therapy was highly sensitive to the timing of drug administration. The optimal treatment protocol was found to consist of recombinant IL-12 given first, followed by an IL-6 inhibitor, followed by anti-PD-1 immunotherapy, with the administration of each drug occurring several days apart. The time delay between treatments reflects a crucial window that is required for each drug to sufficiently alter the tumor micro-environment into a setting that is more conducive to treatment response. The duration of these drug-induced changes was not investigated in this work, therefore further analysis would be required to determine whether they are semi-permanent or more transient in nature. It would also be interesting to investigate the effects of a combination therapy that includes recombinant IFN*γ*, which is expected to overall have a stimulatory effect on the immune response, inducing CD4+ Th1-related changes^27^. While administration of recombinant IFN*γ* has previously resulted controversial clinical findings when combined with first-line chemotherapeutic agents^40^, its potential positive effects would be expected to be robust due to its long half-life. It would be interesting to determine whether a specific phenotype responds best to this combination and whether this response is sensitive to the timing of drug administration.

Lastly, we analyzed a comprehensive ex-vivo data set to determine whether there were any statistically significant differences between responder and non-responder phenotypes in the experimentally measured features that were not captured by the systems biology model. During the clustering of the ex-vivo data into different response phenotypes, the responders were assumed to express higher average granzyme and perforin levels than the non-responders, see Supplementary Section F. Subsequent statistical analysis of the predicted response phenotypes indicated that the average IFN*γ* expression was higher in the predicted responder group. Interestingly, the Ki-67 isotype values were also found to be significantly higher in the predicted responder phenotype under all treatment conditions. This result parallels recent clinical findings for patients with NSCLC treated with anti-PD-1 immunotherapy, in which a higher Ki-67 expression in circulating CD8+ T-cells was seen to predict superior durable clinical benefit and survival rate^35,36^. Furthermore, clinical findings imply that this proliferative immune response following anti-PD-1 immunotherapy is a tumor-specific, rather than non-specific, response^34,35^.

While response to anti-PD-1 immunotherapy is associated with increased Ki-67 expression among CD8+ T-cells, high baseline values of Ki-67 under control conditions could be indicative of a highly proliferative, more aggressive cancer. Indeed, anti-PD-1 immunotherapy has shown unprecedented success for the treatment of several aggressive and hard-to-treat cancers, such as advanced stage melanoma and NSCLC^41^. Interestingly, high Ki-67 expression in breast cancer tissue was also a significant predictive factor for the response to neoadjuvant chemotherapy, especially in ER-negative and HER2-positive breast cancer patients^42^. Importantly, we see from Table 1 that the cancer proliferation rate was identified as one of the top five tumor micro-environment features by all three feature selection techniques, using simulated clinical trial data that was obtained from the systems biology model.

It is interesting that only the machine learning techniques, and not the MPSA method, identified the proliferation rate of cytotoxic CD8+ T-cells as an important feature for distinguishing between response groups, see Table 1. Importantly, while granzyme, perforin, and Ki-67 expression were not directly included in the systems biology model, the simulated clinical trial data indirectly reflected the importance of these quantities in the responder group. For example, the kinetic parameter that was identified as most significant for distinguishing between response and non-response phenotypes, see Table 1, is expected to be related to granzyme and perforin expression. Moreover, the proliferation rate of cytotoxic CD8+ T-cells is related to the total Ki-67 expression level. Thus, we see that by combining systems biology and machine learning approaches, there is a potential to indirectly identify important features that are beyond the scope of the model. Notably, the observed differences in IFN*γ* and Ki-67 expression between responder and non-responder phenotypes are emergent results, since both IFN*γ* and Ki-67 were not used for the phenotype clustering. This finding implies that the clustering approach developed here, built on patient-derived ex-vivo data, has the potential to predict the clinical outcome. Moreover, while the clustering was performed independently of the development of the systems biology model, the importance of IFN*γ* expression was captured by the model and also reflected in the novel triple combination therapy developed in this work, see Table 2 and Fig. 4b. These results illustrate the importance of unbiased simulations and statistical analysis in cancer research, and that such approaches may lead to findings that are crucial for developing efficient and highly effective cancer treatments.

The findings in this study highlight how a combination of systems biology and machine learning approaches can be leveraged to answer a number of questions related to how patient-specific biology modulates immune response. We emphasize that while the current work focuses on several questions related to improving cancer therapies, the approaches developed here are generic and can be applied to other diseases and conditions associated with immune dysfunction, such as diabetes mellitus^43^. Importantly, the approaches developed in this work enable one to identify more targeted experiments for predicting patient response phenotype, thus potentially laying the foundation for the development of cost effective precision drug screening. This work is a proof-of-concept in this direction. Importantly, our results highlight that while some interactions/effects may not be captured by the systems biology model, the machine learning approach can effectively capture them by retraining on the clinical data during the transfer learning step. Furthermore, we anticipate that several refinements can be made to the methodology to more accurately determine the significance of differences in patient-specific biology and to improve the patient response phenotype prediction accuracy without considerably increasing the complexity of the systems biology model.

For example, the approach developed in this work relied on parameter sets that were sampled from uniform distributions and the patient age and sex were not included. However, preliminary statistical analysis of ex-vivo data indicates that there are indeed age and sex-specific differences in the distributions of cytokine expression and T-cell populations, and that the values of these features were normally or log-normally distributed. Incorporating the appropriate distributions, as well as patient demographics, such as age and sex, into the generation of the simulated clinical trial data and the SBINN would therefore be expected to further improve the accuracy of the approach, without adding complexity to the systems biology model. Importantly, this refined methodology, which will be investigated in a future work, incorporates additional patient-specific information while still enabling the development of more cost-efficient, targeted drug-screening experiments.

It is clear from this work that a combination of systems biology and machine learning approaches is crucial for interpreting and learning from small clinical data sets. Particularly, such an integrative approach is imperative when the dimensionality of the experimental/clinical data exceeds the number of samples (patients). In this limit, standard techniques cannot be used to extract important features of response to treatment or to make predictions directly from the clinical data- tasks that are necessary for identifying potential drug resistance mechanisms and for developing more targeted drug-screening experiments. In the approach developed in this work, systems biology was integrated with machine learning algorithms for feature extraction and also for classification of patient response phenotypes using transfer learning. Importantly, we point out that while neural networks were used in this work for classification, it would be interesting to investigate how transfer learning can be combined with other machine learning classification approaches, such as random forest classifier, to improve the prediction of patient response phenotypes. As a preliminary investigation, we performed 10-fold cross validation using the tumor micro-environment features as inputs into a random forest classifier and found that it performed slightly better on the ex-vivo data without transfer learning than the optimal neural network without transfer learning. This begs the question of how significantly the transfer learning approach developed in this work can improve the classification results of other machine learning algorithms for small clinical data sets, which will be investigated in future work.

Another possible extension of this work would be to characterize sub-phenotypes in the two response groups. Here, we defined ‘responder’ as a patient whose tumor size at *t* = 72 h after treatment was less than or equal to the initial tumor size before treatment. However, it can be anticipated that in the ‘non-responder’ category there will be several sub-phenotypes. For example, for some patients, the treatment may have zero effect, thus their tumor will grow at its normal rate. For others, the treatment may have a negative effect and may accelerate tumor growth. In a future work, we will extend this research to the sub-classification of different response phenotypes to understand more about what differentiates them and leverage these findings to improve response to anti-PD-1 immunotherapy.

## Methods

### Experimental protocol

One major factor contributing to the unpredictability of patient response to cancer treatment is the lack of well-established translational platforms that capture the inter- and intra-patient tumor heterogeneity, tumor and stromal cell biology, underlying three-dimensional architecture of the tumor micro-environment, and the tumor-immune cell interactions^44^. Standard in-vitro, in-vivo, and ex-vivo models using cell lines, spheroids, or organotypic tumor models are limited in their ability to capture these biologically relevant features and interactions at the individual patient level, which leads to poor predictability of clinical outcomes. In this work, we used data obtained from an ex-vivo human system^45^, that incorporates fragments from tumor biopsies in co-culture with patient-matched peripheral immune cells and plasma. Tumor biopsies from 50 patients with several stages of head and neck squamous cell carcinoma (HNSCC) were explanted into a culture well containing tumor matrix proteins matching the grade and stage of the tumor type. This platform preserves the patient tumor cellular architecture and heterogeneity with a high degree of morphologic and kinase signaling fidelity^45^, enabling research into the pharmacodynamics of PD-1 inhibitors using a live human system. All experimental protocols and statistical and bioinformatics analysis are explained thoroughly in a previous work. Below we give a summary of the experimental data set and refer the reader to Smalley et al.^27^ for more details.

In experiments, nivolumab (132 μg/mL dose) was administered to the ex vivo human cultures at time *t* = 0, 24, 48 h, with washout and replacement of media between doses. Cytokine levels (IL-4, IL-6, IL-10, IL-12, IFN*γ*) were measured using Luminex cytokine assays for control and treatment conditions at *t* = 24, 48, 72 h, and relative immune cell populations (CD4+ helper T-cells, naive CD8+ T-cells, and cytotoxic CD8+ T-cells) were measured using flow cytometry at *t* = 0, 72 h. The antibodies used in experiments, as well as a figure exemplifying the gating strategy, are presented in Smalley et al.^27^. We note that the data set was not complete for all 50 patients (i.e. human cultures) in the study. We included all available measurements from the 50 patients into the sampling ranges for the generation of simulated clinical trials, see the Systems biology approach: simulated clinical trials section. However, we excluded patients from the phenotype classification tasks if they were missing experimental measurements (such as cytokine levels or T-cell populations) that were necessary for phenotype classification, see Machine learning protocol: classification neural network and Supplementary Section F. Removing these patients reduced the size of the ex-vivo data set to 37 patients. Importantly, it should be noted that the available data for the excluded patients typically fell within the ranges exhibited by the remaining 37 patients, thus we do not expect this to markedly bias the phenotype classification results.

The average patient age in the total data set was 52 ± 13 years, and it was 53 ± 12 years in the reduced (classification) ex-vivo data set with 37 patients. In both cases, the patient tumor stage ranged from T3N0M0 to T4N2bM0 (no patients in the study had distant metastases). In the total data set, 29 patients were female, 20 male, and 1 of unknown sex, while the classification data set was comprised of 19 females, 17 males, and 1 patient with unknown sex. Patient control measurements revealed that the tumor micro-environments were highly heterogeneous, with cytokine expression and immune cell population fractions observed in the following ranges: IFN*γ* 0.18–482.31 pg/mL, IL-12 1.82–11.44 pg/mL, IL-6 149.15–35884.0 pg/mL, IL-4 0.10–61.37 pg/mL, naive CD8+ T-cell fraction 0.21–0.97, cytotoxic CD8+ T-cell fraction 0.0–0.59, and CD4+ helper T-cell fraction 0.01–0.69.

### Systems biology approach: overview

The ex-vivo human experiments indicated that Th1-related changes to the tumor micro-environment have the greatest impact on patient-to-patient heterogeneity resulting from PD-1 blockade^27^. Guided by these results and relevant interactions in the literature, we constructed a multi-scale systems biology (SB) model comprised of a tumor cell population along with five key interacting T-cell populations and four key cytokines involved in T-helper cell differentiation and activation, see Fig. 1. Specifically, the model consists of 17 coupled ordinary differential equations (ODEs), which describe the time evolution of naive CD4+ helper T cells (Th0), type 1 helper T cells (Th1), type 2 helper T cells (Th2), naive CD8+ T cells (TN8), cytotoxic CD8+ T cells (Tc), a cancer cell population, cytokine levels, PD-1 and PD-L1 levels, and drug concentration. The full details of the model, including all the molecular interactions and the corresponding system of coupled ODEs describing the time evolution of each chemical species, are presented in Supplementary Section A. In brief, the main interactions comprising the model are as follows. Cell proliferation and natural death are assumed for all cell populations in the model. Additionally, the CD4+ Th0 cells can differentiate into either CD4+ Th1 cells or CD4+ Th2 cells. The first differentiation process is mediated by the cytokines IL-4 and IL-6. IL-12 and IFN*γ* mediate the second differentiation process. Naive CD8+ cells differentiate into CD8+ Tc cells in the presence of CD4+ Th1 cells, and the proliferation rate of CD8+ Tc cells is increased by IL-12 expression. CD8+ Tc cells kill the cancer cells. All activated T-cells (CD4+ Th1, CD4+ Th2, CD8+ Tc) express PD-1 and PD-L1. Cancer cells also express PD-L1, which is mediated by IFN*γ* expression. PD-1 and PD-L1 form a protein complex which inhibits all T-cell differentiation processes. The production of cytokines depends on the T-cell populations and cancer cell population, and there are several feedback loops which affect the production rates. The resulting pathway, depicted in Supplementary Fig. 1, exhibits several potential mechanisms of intrinsic drug resistance.

### Systems biology approach: estimation of nominal parameter set

**Fig. 6:**
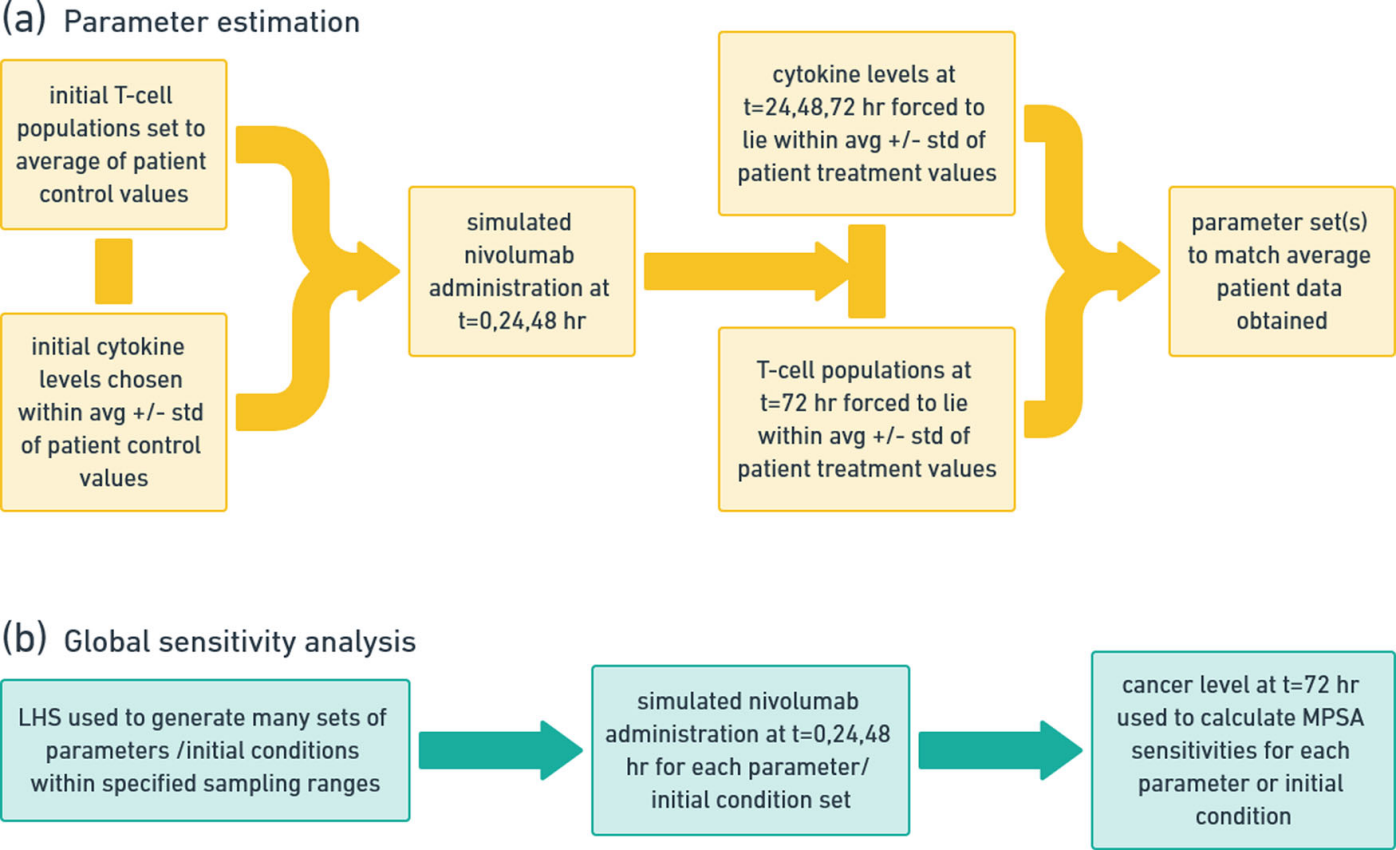
The computational pipeline used to: **a** determine nominal parameter sets by matching to the average patient data and **b** use Latin hypercube sampling (LHS) to perform global sensitivity analysis and simulate a large patient clinical trial.

The human ex-vivo data, including initial concentrations and the dynamic range and changes of different Th1-related cell types and cytokines, were integrated into the systems biology model to determine a nominal parameter set, see Fig. 6a. First, the initial cytokine levels and T-cell populations used in numerical simulations were sampled from the patient (control) data. Then the MATLAB genetic algorithm with the ode15s solver was used to integrate the system of coupled ODEs, where the kinetic parameter ranges were set to previously reported biologically relevant ranges when possible (see Supplementary Section B for details). To simulate the 72-h experimental treatment protocol on the model system, we administered nivolumab treatment at *t* = 0, 24, 48 h. Drug washout between subsequent doses of nivolumab treatment was simulated by setting the free drug level to zero immediately before administering the next dose. The objective function was coded with the constraint that over the 72-hour treatment window, the simulated cytokine expression levels must fall within the range set by the average ±1 standard deviation of the patient data (with nivolumab treatment) at times *t* = 24, 48, 72 h. Similarly, the T-cell populations were forced to lie between average ±1 standard deviation at *t* = 72 h (with nivolumab treatment). Using this approach, it is conceivable that there could be many sets of parameters that fit the average patient data. The nominal parameter set that we obtained is presented in Supplementary Table 3.

Importantly, this nominal parameter set led to a ‘responder’ phenotype. In this work, we define ‘responder’ as a patient whose tumor size at *t* = 72 h after treatment is less than or equal to the initial tumor size before treatment. However, it can be anticipated that in the ‘non-responder’ category there will be several sub-phenotypes. For example, for some patients, the treatment may have zero effect, thus their tumor will grow at its normal rate. For others, the treatment may have a negative effect and may accelerate tumor growth. Investigation of the hierarchy of these sub-phenotypes is left for a future work. However, given the variability in patient response to nivolumab treatment that was observed in the human ex-vivo experiments^27^, we aimed to capture such variability and understand more about the putative mechanisms of intrinsic drug resistance. To this end, we previously performed both local and global sensitivity analysis^27^. Importantly, we found that small perturbations to a single kinetic parameter and/or initial condition via local sensitivity analysis of the model were not sufficient to change the nature of the response to the treatment, i.e. to induce a ‘non-responder’ phenotype. Induction of a ‘non-responder’ phenotype was accomplished via global sensitivity analysis, see Fig. 6b, which involved randomly changing all the initial cytokine levels and initial T-cell populations and/or the values of the kinetic parameters of the model simultaneously to generate virtual patients in a large simulated clinical trial.

### Systems biology approach: simulated clinical trials

The statistically independent parameter sets and initial conditions representing individual virtual patients in the simulated clinical trials were generated efficiently by using the Latin hypercube sampling method^46^. The ranges used for sampling the kinetic parameters, initial cytokine levels, and the initial T-cell populations are presented in Supplementary Table 3. For the initial cytokine levels and T-cell populations, the ranges were set by the lowest and highest values expressed in the untreated (control) human ex-vivo patient data. The ranges for the kinetic parameters were obtained from the literature when possible and estimated when not. The 72-h treatment protocol was simulated for each set of initial conditions and kinetic parameter values, generating a simulated clinical trial patient data set. Typically, simulated clinical trials consisted of at least 200,000 samples (i.e. virtual patients).

Several techniques were used to analyze data from simulated clinical trials and to elucidate which patient features contributed most to the variability in response to treatment. The multi-parametric sensitivity analysis (MPSA) method^47–50^ was used to evaluate sensitivities based on Kolmogorov–Smirnov statistics, returning sensitivity values between 0 and 1, where the reference output was taken to be the size of the cancer cell population at *t* = 72 h obtained with the nominal parameter set. In addition, several machine learning feature selection algorithms were applied to the simulated clinical trial data, as discussed further in Machine learning protocol: feature selection. Using the resulting identified important features along with insights from the systems biology model, we also determined potentially significant resistance nodes.

Simulated clinical trial data was also used to pre-train the neural networks for the transfer learning step, described further in the Machine learning protocol: non-linear regression neural network and Machine learning protocol: classification neural network sections. Importantly, we note that due to biological and tumor heterogeneity, the sampling ranges for initial conditions and kinetic parameter values span several orders of magnitude in most cases. For this reason and given the number of initial conditions and kinetic parameters in the model, as well as the sampling method, it is unlikely that any virtual patient (represented by one sample of initial conditions and kinetic parameter values) precisely corresponds to any specific patient in the ex-vivo data set. Thus, the simulated clinical trial can be considered an external dataset and the risk for overfitting in the transfer learning step (see Machine learning protocol: classification neural network) is negligible.

### Systems biology approach: triple combination therapy

We simulated triple combination treatment to overcome the mechanisms of drug resistance determined via the simulated clinical trials. Specifically, to improve patient response, the anti-PD-1 immunotherapy protocol was given in combination with an IL-6 inhibitor and recombinant IL-12. The equations used to describe the time evolution of the IL-6 inhibitor and recombinant IL-12 concentrations, as well as their effects on the pathway, are presented in Supplementary Section C. The IL-6 inhibition was modeled after the effects of the drug siltuximab, which is given intravenously and has a mean half-life of 20.6 days^51^. Siltuximab works by directly binding to IL-6, preventing it from binding to IL-6 receptors and exerting its immunomodulatory effects. Since the drug reduces the available functional IL-6 level, we modeled siltuximab to directly decrease the IL-6 production rate in a concentration-dependent manner, see Supplementary Section C. For recombinant IL-12, the drug half-life was measured to be ~30 h^52^ in patients after a single subcutaneous injection. For doses in the range of 5–20 μg, the plasma concentration was observed to exceed 10 pg/mL, reaching as high as ~100 pg/mL^53^. These drug pharmacokinetics were taken into account when modeling the administration of recombinant IL-12.

### Statistical analysis

A two-sample Kolmogorov–Smirnov (KS) test and Mann–Whitney *U* (MWU) test (with two-tailed *p*-value) were used to test for statistically significant differences between the response groups in the ex-vivo data set. Both tests were applied to all experimentally measureable features and the patient ages. Fisher’s exact test was also applied to the patient age and sex data to determine if there was a nonrandom association between these patient demographics and response to anti-PD-1 immunotherapy. For the Fisher’s exact test on patient ages, the contingency table was constructed by considering the number of patients above and below a given threshold age (30, 40, 45, 50, 55, 60, 65, and 70 years) in each response group. Spearman’s *ρ* was calculated to determine the correlation coefficient between paired samples in simulated clinical trial data. The non-parametric MWU test (with one-tailed *p*-value) was also used to test for statistically significant differences between the neural network phenotype classification results obtained with and without transfer learning.

### Machine learning protocol: overview

Several machine learning techniques were utilized in this study to analyze and make predictions about both the ex-vivo data set and the simulated clinical data sets obtained using the systems biology model. All feature selection techniques and clustering algorithms were implemented in MATLAB version R2018a^54^. All neural network calculations were implemented in Python version 3.6.9, using the Keras Deep Learning Library with TensorFlow backend^55^. For all neural networks, the Adam^56^ optimizer was used to update the network weights, with the learning rate and decay chosen to optimize the network performance. The network inputs were standardized using the Python scikit-learn StandardScalar function^57^ and unless otherwise specified, the default Keras settings were used for both neural networks. The backpropagation algorithm was used during training of neural networks. Bayesian optimization was used to tune the learning parameters and network hyperparameters, as outlined in Supplementary Section E.

### Machine learning protocol: feature selection

Fisher discriminant analysis^58^ and simple filter feature selection methods^59,60^ were applied to the simulated patient data set to infer which kinetic parameters and experimentally measurable cytokine levels and T-cell populations were most important for segregating response phenotypes. For the filter feature selection technique, 10-fold cross validation was used, where the simulated data was split into training and testing groups with a ratio of 3:1. For Fisher discriminant analysis, the simulated data was split into training and testing groups with a ratio of 1:1. Fisher discriminant analysis was repeated 100 times, and for each run, features were ranked from highest to lowest based on the standardized canonical coefficients. The rankings for each run were summed, and the features with the highest total ranking after 100 runs were reported.

The purpose of applying feature selection in this work was twofold. First, identifying these important features is necessary for developing more targeted drug-screening experiments and for identifying potential mechanisms of drug resistance. Second, we wanted to probe whether selecting features from simulated clinical trials (and applying transfer learning) produces classification results that are at least as accurate as when the features are selected directly from clinical data. The point of this is to illustrate that simulated data can be used to supplement small clinical data sets when the feature space exceeds the number of samples, in which case feature selection techniques cannot be directly applied to the clinical data. To this end, Fisher discriminant analysis was also applied directly to the ex-vivo dataset as part of the learning algorithm, for each step of 10-fold cross validation, as explained further in the Machine learning protocol: classification neural network section.

### Machine learning protocol: non-linear regression neural network

Patient-specific kinetic parameter values were calculated using a non-linear regression deep neural network^61,62^, which we refer to as the NR-SBINN. This was accomplished by training the NR-SBINN on simulated patient data, then directly applying the pre-trained network to the ex-vivo patient data set. The simulated patient data used for training the network was generated as follows. First, a clinical trial of 200,000 patients was simulated using the systems biology model, wherein only the parameter value and the initial cytokine and T-cell populations were altered, while the other kinetic parameters were fixed at their nominal values, see Supplementary Section B. LHS was used to generate the statistically independent samples of the kinetic parameter value and initial conditions. The Pearson correlation coefficient was calculated between the kinetic parameter and the experimentally measureable cytokine levels and T-cell populations, and correlations were found to be highest with IFN*γ* expression at all measured time points. The IFN*γ* expression values, specifically the IFN*γ* levels at *t* = 0, 24, 48, and 72 h, were then used as the inputs into the NR-SBINN. To improve the time to convergence and the network accuracy, an additional simulated clinical trial was then generated in which the correlations between the kinetic parameter and the initial IFN*γ* expression were artificially inflated during the LHS step. This simulated clinical trial was ultimately used for training the NR-SBINN, which enabled the identification and the enhancement of important correlations between the parameter value and experimentally measurable features, allowing the NR-SBINN to obtain a high prediction accuracy.

The learning parameters and network hyperparameters were tuned to achieve optimal performance of the NR-SBINN. The tuning method and the optimal NR-SBINN architecture, including the network hyperparameters and learning parameters, are discussed in Supplementary Section E. During training of the optimal NR-SBINN, the simulated clinical data was split into training and testing sets with a ratio of 99:1. In addition, 5% of the training set was used for validation during training. The loss function of the NR-SBINN was taken to be the mean-squared error. With the optimal network hyperparameters and learning parameters, the NR-SBINN converged with high accuracy on the simulated data after a small number of training epochs, see Supplementary Fig. 3. The trained NR-SBINN was then applied to the ex-vivo data set to predict the patient-specific kinetic parameter value directly from the ex-vivo IFN*γ* expression measurements.

### Machine learning protocol: classification neural network

A classification deep neural network^61,62^, which we refer to as C-SBINN, was developed to predict the patient response phenotype based on the results of the feature selection techniques. For the transfer learning protocol, the C-SBINN was first trained on an imbalanced simulated patient data set, which was randomly sampled from the simulated clinical trial (see Systems biology approach: simulated clinical trials) to have a distribution of 22% responders and 78% non-responders to reflect the class imbalance in the ex-vivo data set, see Supplementary Section F. During training, the Python scikit-learn compute_class_weight function with the “balanced” argument was used to mitigate the class imbalance. The binary cross-entropy error was taken as the loss function. For this training step, the simulated clinical data was split into training and testing sets with a ratio of 99:1 and 10% of the training data was reserved for validation. Next, transfer learning^28^ was implemented, where the pre-trained C-SBINN was re-trained on a subset of the ex-vivo data to improve its prediction accuracy on this data. The ex-vivo training and testing sets were randomly sampled from the entire ex-vivo data set, subject to the constraint that the class imbalance exhibited by the entire data set was approximately preserved (training sets consisted of 28 patients, testing sets were 9 patients). To compare the improvement in prediction accuracy resulting from transfer learning, a classification deep neural network was also separately trained only on the ex-vivo data, where the training and testing sets were identical to those used in the transfer learning approach. Tenfold cross validation was used to validate the results. In all cases, the input features were standardized using the Python scikit-learn StandardScaler. To ensure there was no data leakage, for each cross validation fold, the StandardScaler was fit only to the training set and then applied to the testing set.

In this work, we compared the performance of classification neural networks for several different sets of input features that were determined by the feature selection techniques described in Machine learning protocol: feature selection. Using the simulated clinical data, the feature selection techniques identified a set of features that predominantly characterized the tumor micro-environment, as well as a set of features that characterized the response dynamics to anti-PD-1 immunotherapy. For comparison, we also used input features that were determined directly from the ex-vivo data set. In the latter case, we performed 10-fold cross validation, and for each step, we randomly selected a training and testing set that preserves the distribution of responders and non-responders exhibited by the entire ex-vivo data set. For each cross validation step, we then performed Fisher discriminant analysis on the training set, selected the top six experimental features, and then used those features as inputs into the neural network.

Importantly, the learning parameters and network hyperparameters were tuned separately for each learning approach and set of input features to identify an optimal neural network architecture for each case. The tuning method and the optimal network architectures for all sets of input features and learning approaches, including network hyperparameters and learning parameters, are discussed in Supplementary Section E. For each case, the optimal network was implemented to ensure a fair comparison between approaches and input features (see Supplementary Section E for more details).

### Machine learning protocol: performance metrics

The accuracy of the NR-SBINN was measured by computing the average relative prediction error and associated standard deviation over all data points in the testing set. For the C-SBINN, the accuracy of the network was measured by computing several performance metrics, including: the Cohen kappa score (CKS)^30,63^, the Matthew correlation coefficient (MCC)^64^ (shown to be the best measure of classification accuracy for imbalanced data sets^65^), the area under the receiving-operator characteristics (ROC) curve^66^, and the area under the precision-recall curve (PRC)^67–69^ (the latter of which has also shown better performance for imbalanced data sets). The averages and standard deviations of the performance metrics were computed over the 10-fold cross validation. The ROC curves and precision-recall curves were computed using the Python scikit-learn functions^57^. The G-mean^70^ and the F1-score^71,72^ were calculated and used to adjust the classification probability threshold^73^ to maximize the MCC for the imbalanced data sets. A summary of the terminology and the procedures for calculating each performance metric is presented in Supplementary Table 4.

Lastly, in order to train and test the C-SBINN on the ex-vivo patient data, it was necessary for each sample to be labeled. In the absence of patient-matched clinical outcome, we developed a statistical approach that used *k*-means clustering^74^ with the ex-vivo expression of granzyme and perforin, which are released by cytotoxic T-cell lymphocytes and natural killer cells to induce apoptosis in target cells, such as cancer cells. The full details of the ex-vivo labeling approach are presented in Supplementary Section F.

### Reporting summary

Further information on research design is available in the Nature Research Reporting Summary linked to this article.

## Supplementary information

Supplementary Information

Description of additional supplementary files

Supplementary Data 1

Supplementary Data 2

Supplementary Data 3

Supplementary Data 4

Reporting Summary

## Data Availability

The datasets generated during and/or analyzed during the current study are available from the corresponding author on reasonable request.

## Code availability

Custom code related to simulated treatment protocols for the ex-vivo experiments with specified inputs and outputs, as well as comments throughout the code, can be found at https://github.com/mprzedborski/ex-vivo-PD1-blockade^75^.
