# Supplementary material for: Systems biology informed neural networks (SBINN) predict response and novel combinations for PD-1 checkpoint blockade
Source: Commun Biol. 2021 Jul 15;4:877. doi: 10.1038/s42003-021-02393-7 (PMC8282606; doi:10.1038/s42003-021-02393-7)
Supplement: Supplementary file 1 — Supplementary Information [file 42003_2021_2393_MOESM1_ESM.pdf]

Supplementary Information for: Systems biology informed neural networks (SBINN) predict response and novel combinations for PD-1 checkpoint blockade

Michelle Przedborski[1,*], Munisha Smalley[2], Saravanan Thiyagarajan[2], Aaron Goldman[3,4], Mohammad Kohandel[1]

[1]Department of Applied Mathematics, University of Waterloo, Waterloo, ON, Canada
[2]Integrative Immuno Oncology Center, Mitra Biotech, Woburn, MA, USA
[3]Division of Engineering in Medicine, Brigham and Women's Hospital, Boston, Massachusetts, USA
[4]Department of Medicine, Harvard Medical School, Boston, Massachusetts, USA
[*]Corresponding author: mprzedborski@uwaterloo.ca

A Mathematical model

Given the experimental measurements of cytokine expression and relative T-cell populations in ex-vivo human tumor cultures, and taking into account well-established immune cell interactions in the literature, we developed the interaction network depicted in Supplementary Figure 1. An explanation of each of the cellular and protein species appearing in Supplementary Figure 1 is presented in Supplementary Table 1, along with its variable representation in the mathematical equations.

Table 1: Cellular and protein species in the interaction network in Supplementary Figure 1 of the manuscript.

**Cellular species**	**Description**	**Mathematical representation**
CD4+ Th0	Naive helper (CD4+) T-cell population	T_{N4}
CD4+ Th1	Type 1 helper T-cell population	Th_1
CD4+ Th2	Type 2 helper T-cell population	Th_2
Naive CD8+	Naive cytotoxic (CD8+) T-cell population	T_{N8}
CD8+ Tc	Cytotoxic (CD8+) T-cell population	T_c
DC	Dendritic cell population	–
Cancer	Cancer cell population	C
**Protein species**	**Description**	**Mathematical representation**
IL-4	Concentration of interleukin 4	$[IL\text{-}4]$
IL-6	Concentration of interleukin 6	$[IL\text{-}6]$
IL-12	Concentration of interleukin 12	$[IL\text{-}12]$
IFNγ	Concentration of interferon gamma	$[IFN\gamma]$
PD-1	Concentration of programmed cell death protein 1	$[PD\text{-}1]$
PD-L1	Concentration of programmed death-ligand 1	$[PD\text{-}L1]$
PD-1:PD-L1	Concentration of PD-1:PD-L1 protein complex	$[PD\text{-}1{:}PD\text{-}L1]$
**Drug species**	**Description**	**Mathematical representation**
Nivolumab	Concentration of the PD-1 inhibitor Nivolumab	$[A]$
–	Concentration of Nivolumab:PD-1 complex	$[A : PD\text{-}1]$

Below we explain each interaction in the network, along with its mathematical formulation, in addition to the key assumptions in the model. For each cellular species, it is assumed that cells proliferate via mitosis and die at rates that are proportional to their population size.

1. Time evolution of naive helper (CD4+) T-cell population:

$$\frac{dT_{N4}}{dt} = n_4 T_{N4} - \left(d_{1\text{-}12} T_{N4} \frac{[IL\text{-}12]}{q_{dIL12} + [IL\text{-}12]} + d_{1\text{-}IFN} T_{N4} \frac{[IFN\gamma]}{q_{IFN\text{-}1} + [IFN\gamma]} \right) \left(\frac{s_1}{s_1 + [PD\text{-}1 : PD\text{-}L1]} \right) - \left(d_2 T_{N4} \frac{[IL\text{-}4]}{q_{dIL4} + [IL\text{-}4]} \right) \left(\frac{s_2}{s_2 + [PD\text{-}1 : PD\text{-}L1]} \right) \tag{1}$$

The first term describes the net proliferation of T_{N4} cells. The next two terms describe the differentiation of T_{N4} cells into Th_1 cells in the presence of IL-12 [1] (term 2) and IFNγ [2] (term 3). Both of these differentiation processes are inhibited by the PD-1:PD-L1 complex [3–5]. The last term describes the differentiation of T_{N4} cells into Th_2 cells in the presence of IL-4 [1,2], which is inhibited by the PD-1:PD-L1 complex [3–5].

2. Time evolution of type 1 helper T-cell population:

$$\frac{dTh_1}{dt} = n_1 Th_1 + \left(d_{1\text{-}12} T_{N4} \frac{[IL\text{-}12]}{q_{dIL12} + [IL\text{-}12]} + d_{1\text{-}IFN} T_{N4} \frac{[IFN\gamma]}{q_{IFN\text{-}1} + [IFN\gamma]} \right) \left(\frac{s_1}{s_1 + [PD\text{-}1 : PD\text{-}L1]} \right) \tag{2}$$

The first term describes the net proliferation of Th_1 cells. The remaining terms describe the increase in the Th_1 cell population due to the differentiation of T_{N4} cells into Th_1 cells in the presence of IL-12 [1] (term 2) and IFNγ [2] (term 3), which is inhibited by the PD-1:PD-L1 complex [3–5].

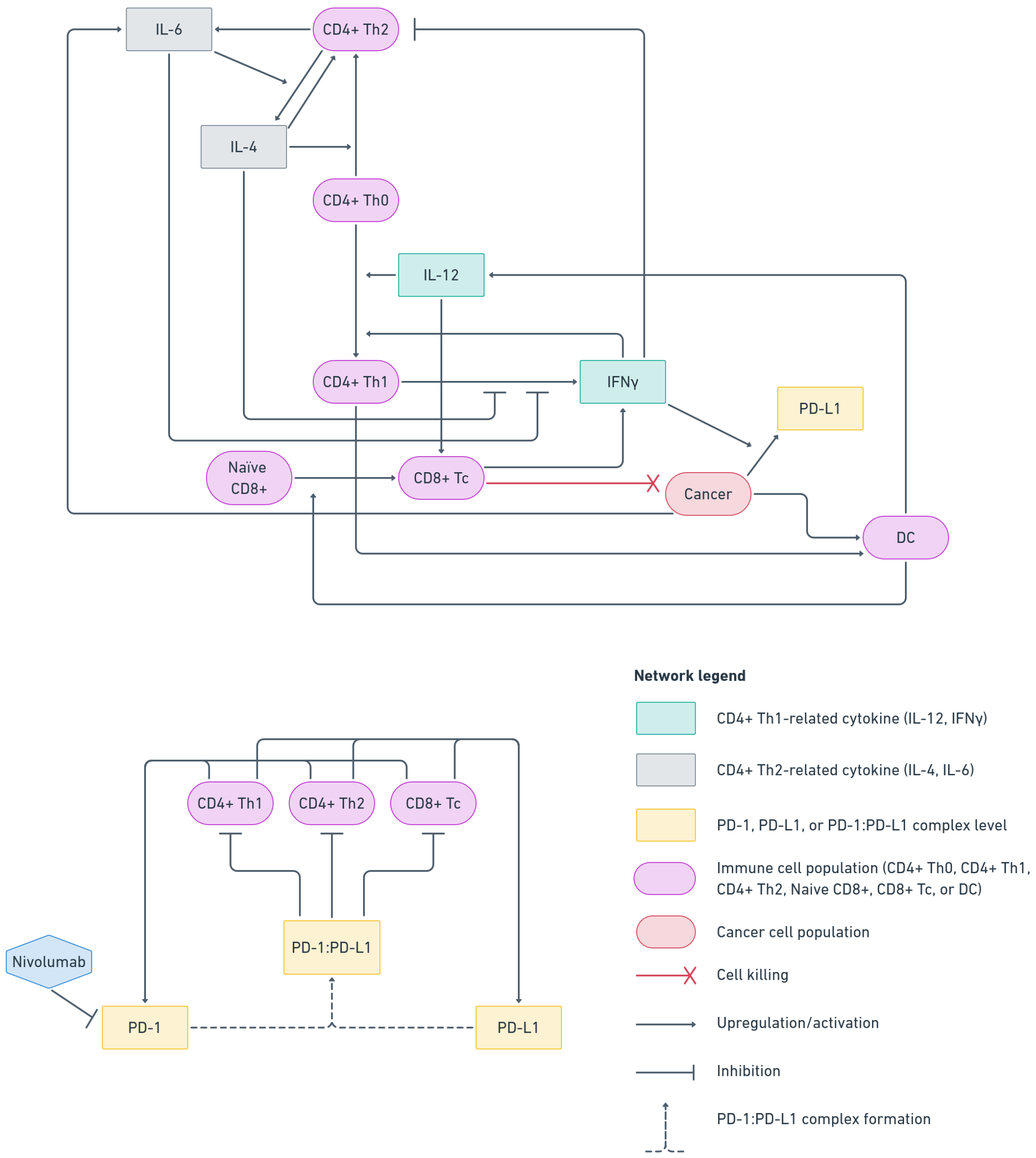

Figure 1: The molecular pathway developed for the SB approach, grounded on experimental data and molecular and cellular interactions from the literature.

3. Time evolution of type 2 helper T-cell population:

$$\frac{dTh_2}{dt} = \left(g_2 Th_2 + g_{2\text{-}4} Th_2 \frac{[IL\text{-}4]}{q_{gIL4} + [IL\text{-}4]}\right)\left(\frac{r_{IFN}}{r_{IFN} + [IFN\gamma]}\right) + \left(d_2 T_{N4} \frac{[IL\text{-}4]}{q_{dIL4} + [IL\text{-}4]}\right)\left(\frac{s_2}{s_2 + [PD\text{-}1 : PD\text{-}L1]}\right) - \delta_2 Th_2 \quad (3)$$

The first term describes the proliferation of Th_2 cells due to mitosis, which is upregulated by IL-4 [1,2] (term 2). However, this cell proliferation is inhibited by IFNγ [6]. The next term describes the increase in Th_2 population resulting from the differentiation of T_{N4} cells into Th_2 cells in the presence of IL-4 [1,2] (term 3), which is inhibited by the PD-1:PD-L1 [3–5] complex. The last term describes natural death of the Th_2 cells.

4. Time evolution of naive cytotoxic (CD8+) T-cell population:

$$\frac{dT_{N8}}{dt} = n_8 T_{N8} - d_c T_{N8}\left(\frac{Th_1}{q_1 + Th_1}\right)\left(\frac{s_C}{s_C + [PD\text{-}1 : PD\text{-}L1]}\right) \quad (4)$$

The first term describes the net proliferation of T_{N8} cells. The second term describes the differentiation of T_{N8} cells into T_c cells in the presence of Th_1 [7,8], which is inhibited by the PD-1:PD-L1 complex [3–5].

5. Time evolution of cytotoxic (CD8+) T-cell population:

$$\frac{dT_c}{dt} = n_c T_c + g_{c-12} T_c \frac{[IL\text{-}12]}{q_{gIL12} + [IL\text{-}12]} + d_c T_{N8}\left(\frac{Th_1}{q_1 + Th_1}\right)\left(\frac{s_c}{s_c + [PD\text{-}1 : PD\text{-}L1]}\right) \quad (5)$$

The first term describes the net proliferation of T_c cells, which is upregulated by IL-12 [9] (term 2). The third term describes the increase in the T_c cell population due to the differentiation of T_{N8} cells into T_c cells in the presence of DCs that have been activated by Th_1 [7,8]. This differentiation process is inhibited by the PD-1:PD-L1 complex [3–5].

6. Time evolution of cancer cell population:

$$\frac{dC}{dt} = n_{Can} C - k_c C T_c \quad (6)$$

The first term describes the net proliferation of cancer cells and the second term describes the killing of cancer cells by T_c cells through mechanisms such as granzyme/perforin-induced apoptosis [3,10].

7. Time evolution of IFNγ concentration:

$$\frac{d[IFN\gamma]}{dt} = p_{1\text{-}IFN} Th_1 \left(\frac{r_{IL4}}{r_{IL4} + [IL\text{-}4]}\right)\left(\frac{r_{IL6}}{r_{IL6} + [IL\text{-}6]}\right) + p_{c\text{-}IFN} T_c - \delta_{IFN}[IFN\gamma] \quad (7)$$

Term one describes the secretion of IFNγ by Th_1 cells [1, 2, 6], which is inhibited by IL-4 [11] and IL-6 [2]. The second term describes the secretion of IFNγ by T_c cells [3], and the third term describes the natural decay of IFNγ.

8. Time evolution of IL-4 concentration:

$$\frac{d[IL\text{-}4]}{dt} = p_{2\text{-}4} Th_2 + p_{2\text{-}4\text{-}6} Th_2 \left(\frac{[IL\text{-}6]}{q_{IL6} + [IL\text{-}6]}\right) - \delta_{IL4}[IL\text{-}4] \quad (8)$$

The first term describes the secretion of IL-4 by Th_2 cells [1, 2, 6]. The second term describes the additional secretion of IL-4 by Th_2 cells in the presence of IL-6 [2, 12]. The third term describes the natural decay of IL-4.

9. Time evolution of IL-6 concentration:

$$\frac{d[IL\text{-}6]}{dt} = p_{2\text{-}6} Th_2 + p_{Can\text{-}6} C - \delta_{IL6}[IL\text{-}6] \quad (9)$$

Term one describes the secretion of IL-6 by Th_2 cells [6]. Antigen presenting cells produce IL-6 [2, 13] and we assume that the number of antigen presenting cells is directly proportional to the number of cancer cells (term 2). The third term describes the natural decay of IL-6.

10. Time evolution of IL-12 concentration:

$$\frac{d[IL\text{-}12]}{dt} = p_{Can\text{-}12}C + p_{1\text{-}12}Th_1 - \delta_{IL12}[IL\text{-}12] \tag{10}$$

Term one describes the production of IL-12 by DCs, which we assume to be directly proportional to the number of cancer cells [13]. Term two describes the additional production of IL-12 by DCs that are activated by Th_1 cells [14]. The third term describes the natural decay of IL-12.

11. PD-1 concentration and its time evolution:

$$[PD\text{-}1] = \rho\,(Th_1 + Th_2 + T_c) \tag{11}$$

$$\frac{d[PD\text{-}1]}{dt} = \rho\left(\frac{dTh_1}{dt} + \frac{dTh_2}{dt} + \frac{dT_c}{dt}\right) - \beta_+[PD\text{-}1][PD\text{-}L1] + \beta_-[PD\text{-}1 : PD\text{-}L1] - \alpha_+[PD\text{-}1][A] + \alpha_-[A : PD\text{-}1] \tag{12}$$

PD-1 is expressed on all activated T-cells, i.e. Th_1, Th_2, and T_c [3,5], thus the total concentration of PD-1 is proportional to the sum of the T-cell populations, as indicated in Equation (11). We make the simplifying assumption that the same amount of PD-1 is expressed on all types of T-cells, thus the proportionality constants for each population are the same.

The time evolution of PD-1 is described by Equation (12). The first three terms describe the change in the PD-1 levels due to changing T-cell populations. The fourth term describes the binding of PD-1 to PD-L1 to form the PD-1:PD-L1 complex and the fifth term describes the dissociation of the PD-1:PD-L1 complex [3]. The last two terms describe, respectively, the binding of PD-1 to Nivolumab and the dissociation of the Nivolumab:PD-1 complex [5].

12. PD-L1 concentration and its time evolution:

$$[PD\text{-}L1] = \lambda\,(Th_1 + Th_2 + T_c + C) + \lambda_{Can\text{-}IFN}C\left(\frac{[IFN\gamma]}{q_{IFN\text{-}PDL1} + [IFN\gamma]}\right) \tag{13}$$

$$\frac{d[PD\text{-}L1]}{dt} = \lambda\left(\frac{dTh_1}{dt} + \frac{dTh_2}{dt} + \frac{dT_c}{dt} + \frac{dC}{dt}\right) + \lambda_{Can\text{-}IFN}\frac{dC}{dt}\left(\frac{[IFN\gamma]}{q_{IFN\text{-}PDL1} + [IFN\gamma]}\right) - \beta_+[PD\text{-}1][PD\text{-}L1] + \beta_-[PD\text{-}1 : PD\text{-}L1] \tag{14}$$

PD-L1 is expressed on all activated T cells, i.e. Th_1, Th_2, T_c [3,4] as well as cancer cells [3–5], thus the total concentration of PD-L1 is in part proportional to the sum of the T-cell and cancer cell populations, as indicated by the first four terms in Equation (13). We make the simplifying assumption that the PD-L1 expression is identical for all types of cells. In addition, the expression of PD-L1 by cancer cells is upregulated by IFNγ [3–5], as indicated by the fifth term in Equation (13).

The time evolution of PD-L1 is described by Equation (14). The first five terms describe the change in PD-L1 levels due to changing T-cell and cancer cell populations. We make the simplifying assumption that the proteins reach their steady state values instantaneously with respect to the time scale of the changes in cell populations (i.e. the cell division rate) so that $\frac{d[IFN\gamma]}{dt} \approx 0$. The last two terms describe the binding of PD-1 to PD-L1 and the dissociation of the PD-1:PD-L1 complex [3], respectively.

13. Time evolution of PD-1:PD-L1 complex concentration:

$$\frac{d[PD\text{-}1 : PD\text{-}L1]}{dt} = \beta_+[PD\text{-}1][PD\text{-}L1] - \beta_-[PD\text{-}1 : PD\text{-}L1] \tag{15}$$

The first term describes the binding of PD-1 to PD-L1 and the second term describes the dissociation of the PD-1:PD-L1 complex [3].

14. Time evolution of free Nivolumab concentration:

$$\frac{d[A]}{dt} = \tilde{A}(t) - \alpha_+[A][PD\text{-}1] + \alpha_-[A : PD\text{-}1] - \delta_A[A] \tag{16}$$

The first term describes the introduction of Nivolumab into the system, which may be time-dependent, depending on the treatment schedule. The second term describes the binding of PD-1 to Nivolumab, resulting in the formation of the Nivolumab:PD-1 complex, and the third term describes the dissociation of the Nivolumab:PD-1 complex [5]. We make the assumption that the dissociation constant $K_\alpha \equiv \alpha_-/\alpha_+ \ll K_\beta \equiv \beta_-/\beta_+$ so that Nivolumab has a higher binding affinity for PD-1 than PD-L1 does, which allows the drug to displace PD-L1 from the PD-1:PD-L1 complex. Further, in simulations we assume that the rate of association of PD-1 is equivalent for Nivolumab and PD-L1, i.e. $\alpha_+ = \beta_+$, which removes a kinetic parameter from the system. The fourth term in the equation describes the natural decay of Nivolumab.

15. Time evolution of Nivolumab:PD-1 complex concentration:

$$\frac{d[A:PD\text{-}1]}{dt} = \alpha_+[A][PD\text{-}1] - \alpha_-[A:PD\text{-}1] \tag{17}$$

The first term describes the binding of Nivolumab with PD-1 to form the Nivolumab:PD-1 complex, and the second term describes the dissociation of the drug complex [5].

In Supplementary Table 2 we give a description of the kinetic parameters that appear in the above equations in the mathematical model.

Table 2: Description of the kinetic parameters in the mathematical model.

**Number**	**Name**	**Description**
1	n_4	Net proliferation rate of T_{N4} cells
2	n_8	Net proliferation rate of T_{N8} cells
3	n_1	Net proliferation rate of Th_1 cells
4	n_c	IL12-independent net proliferation rate of T_c cells
5	n_{Can}	Net proliferation rate of cancer cells
6	g_2	IL4-independent growth rate of Th_2 cells
7	$g_{2\text{-}4}$	IL4-dependent growth rate of Th_2 cells
8	$g_{c\text{-}12}$	IL12-dependent growth rate of T_c cells
9	δ_2	Death rate of Th_2 cells
10	$d_{1\text{-}IFN}$	IFNγ-dependent differentiation rate of T_{N4} cells into Th_1 cells
11	$d_{1\text{-}12}$	IL12-dependent differentiation rate of T_{N4} cells into Th_1 cells
12	d_2	IL4-dependent differentiation rate of T_{N4} cells into Th_2 cells
13	d_c	Rate of differentiation of T_{N8} cells into T_c cells
14	k_c	Rate of cancer cell killing by T_c cells
15	$p_{1\text{-}IFN}$	Rate of production of IFNγ by Th_1 cells
16	$p_{2\text{-}4\text{-}6}$	IL6-dependent production of IL-4 by Th_2 cells
17	p_{Can-6}	Rate of production of IL-6 by antigen presenting cells (assumed proportional to the number of cancer cells)
18	p_{Can-12}	Rate of production of IL-12 by DCs (assumed proportional to the number of cancer cells)
19	δ_{IFN}	Decay rate of IFNγ
20	δ_{IL4}	Decay rate of IL-4
21	δ_{IL6}	Decay rate of IL-6
22	δ_{IL12}	Decay rate of IL-12
23	δ_A	Decay rate of Nivolumab
24	q_1	Half-maximal Th_1 cell population for T_{N8} differentiation into T_c cells

25	$q_{IFN\text{-}1}$	Half-maximal IFNγ concentration for IFNγ-dependent differentiation of T_{N4} cells into Th_1 cells
26	$q_{IFN\text{-}PDL1}$	Half-maximal IFNγ concentration for IFNγ-dependent PD-L1 expression by cancer cells
27	q_{gIL4}	Half-maximal IL-4 concentration for IL4-dependent proliferation of Th_2 cells
28	q_{dIL4}	Half-maximal IL-4 concentration for IL4-dependent differentiation of T_{N4} cells into Th_2 cells
29	q_{IL6}	Half-maximal IL-6 concentration for IL6-dependent production of IL-4 by Th_2 cells
30	q_{dIL12}	Half-maximal IL-12 concentration for IL12-dependent differentiation of T_{N4} cells into Th_1 cells
31	q_{gIL12}	Half-maximal IL-12 concentration for IL12-dependent proliferation of T_c cells
32	r_{IFN}	Half-maximal IFNγ concentration for IFNγ-dependent inhibition of Th_2 proliferation
33	r_{IL4}	Half-maximal IL-4 concentration for IL4-dependent inhibition of IFNγ production by Th_1 cells
34	r_{IL6}	Half-maximal IL-6 concentration for IL6-dependent inhibition of IFNγ production by Th_1 cells
35	ρ	Per-cell expression level of PD-1
36	λ	Per-cell expression level of PD-L1
37	$\lambda_{Can\text{-}IFN}$	IFNγ-dependent PD-L1 expression per cancer cell
38	β_+	Rate of association of PD-1 and PD-L1
39	β_-	Rate of dissociation of PD-1:PD-L1 complex
40	α_-	Rate of dissociation of Nivolumab:PD-1 complex
41	s_1	Half-maximal PD-1:PD-L1 concentration for inhibition of T_{N4} differentiation into Th_1 cells
42	s_2	Half-maximal PD-1:PD-L1 concentration for inhibition of T_{N4} differentiation into Th_2 cells
43	s_c	Half-maximal PD-1:PD-L1 concentration for inhibition of T_{N8} differentiation into T_c cells
44	$p_{1\text{-}12}$	Rate of IL-12 production by Th_1 cells
45	$p_{2\text{-}4}$	Rate of IL6-independent production of IL-4 by Th_2 cells
46	$p_{2\text{-}6}$	Rate of IL-6 production by Th_2 cells
47	$p_{c\text{-}IFN}$	Rate of IFNγ production by T_c cells

B Parameter values

In Supplementary Table 3, we present the numerical values of the parameters and initial conditions (protein levels and relative T-cell populations) that match to the average patient data (see "nominal value" column), as well as the corresponding units (see "units" column). We use the abbreviation "min" to denote a timescale of minutes. We also present a range for each parameter which was used for searching the parameter space with Matlab's genetic algorithm to match the average patient data as well as for performing the global sensitivity analysis (see "range" column). The ranges presented for the protein levels and T-cell fractions that were obtained from the patient data are set by the minimum and maximum experimentally measured values for all patients, and were used for the global sensitivity analysis. When using the genetic algorithm to match the average patient data, the T-cell fractions were set to the average of all patients without treatment, and the protein levels were sampled from a range set by the average +/- one standard deviation, as explained in the main text.

We note that parameters 44-47 do not have a specified range since they were calculated at the beginning of each simulation by assuming the initial protein levels are steady state protein levels (Equations 7-10) using the initial T-cell population values, and additionally imposing the constraint that all parame-

ters are non-negative. Thus with the local and global sensitivity analysis, it was necessary to re-calculate parameters 44-47 for each simulation.

To ensure that Nivolumab has a higher binding affinity for PD-1 than PD-L1 does, we also imposed the constraint (parameter 40 $<$ 0.1 parameter 39) for each simulation.

Additionally, we note that the PD-1 and PD-L1 concentrations were initialized for each simulation using Equations 11 and 13, respectively, with the initial cell populations and relevant protein level. Finally we point out that in an initial analysis, we used a larger upper bound for the net proliferation rate of cancer cells, parameter 5. In some cases, this led to nonphysical growth of the cancer population over the three day treatment window when there was no treatment response. In these cases, the model output was most sensitive to parameters controlling the CD8+ cytotoxic T-cell population and its efficiency at killing the cancer cells. We present additional important notes below the table.

Table 3: Values and ranges of the kinetic parameters, initial protein levels, and initial T-cell populations used for local and global sensitivity analysis.

Parameter	Nominal value	Range	Units	Reference
1	2.9×10^{-2}	$\ln(2)/20 - \ln(2)$	day^{-1}	estimated from [6,11]
2	8.2×10^{-3}	$\ln(2)/20 - \ln(2)$	day^{-1}	estimated from [6,11]
3	7.7×10^{-3}	$\ln(2)/20 - \ln(2)$	day^{-1}	estimated from [6,11]
4	8.3×10^{-3}	$\ln(2)/20 - \ln(2)$	day^{-1}	estimated from [6,11]
5	6.9×10^{-2}	$\ln(2)/100 - \ln(2)/5$	day^{-1}	estimated
6	3.7×10^{-2}	$\ln(2)/20 - \ln(2)$	day^{-1}	estimated from [6,11]
7	3.9×10^{-2}	$\ln(2)/20 - \ln(2)$	day^{-1}	estimated from [6,11]
8	3.8×10^{-2}	$\ln(2)/20 - \ln(2)$	day^{-1}	estimated from [6,11]
9	1.2×10^{-2}	$\ln(2)/60 - \ln(2)/7$	day^{-1}	estimated from [6,11]
10	7.4×10^{-2}	$\ln(2)/20 - \ln(2)$	day^{-1}	estimated from [15]
11	7.4×10^{-2}	$\ln(2)/20 - \ln(2)$	day^{-1}	estimated from [15]
12	3.6×10^{-2}	$\ln(2)/20 - \ln(2)$	day^{-1}	estimated from [15]
13	3.7×10^{-2}	$\ln(2)/20 - \ln(2)$	day^{-1}	estimated from [15]
14	3.6×10^{-4}	$10^{-8} - 10^{-2}$	T_c cell$^{-1}\cdot$ day^{-1}	estimated
15	8.9×10^{-4}	$6.5 \times 10^{-4} - 1.7 \times 10^{-2}$	$\frac{\text{pg/mL}}{Th_1 \text{ cell} \cdot \text{day}}$	estimated
16	3.9×10^{-4}	$1.4 \times 10^{-7} - 1.4 \times 10^{-2}$	$\frac{\text{pg/mL}}{Th_2 \text{ cell} \cdot \text{day}}$	estimated
17	8.9×10^{-3}	$7.2 \times 10^{-3} - 7.2 \times 10^{-1}$	$\frac{\text{pg/mL}}{\text{cancer cell} \cdot \text{day}}$	estimated
18	1.3×10^{-6}	$8.3 \times 10^{-7} - 9.7 \times 10^{-6}$	$\frac{\text{pg/mL}}{\text{cancer cell} \cdot \text{day}}$	estimated from [16]
19	7.0×10^{-4}	$\ln(2)/1000 - \ln(2)/60$	min^{-1}	estimated from [11]
20	7.7×10^{-4}	$\ln(2)/1000 - \ln(2)/60$	min^{-1}	estimated from [11]
21	7.0×10^{-4}	$\ln(2)/1000 - \ln(2)/60$	min^{-1}	estimated from [11]
22	4.8×10^{-4}	$\ln(2)/1440 - \ln(2)/600$	min^{-1}	estimated from [16]
23	4.7×10^{-2}	$\ln(2)/15 - \ln(2)/10$	day^{-1}	estimated from [16]
24	1.7×10^{2}	$1 - 10^{5}$	Th_1 cells	estimated
25	8.6×10^{-1}	$10^{-3} - 10^{2}$	$[IFN\gamma]$ (pg/mL)	estimated
26	4.1×10^{-1}	$10^{-3} - 10^{2}$	$[IFN\gamma]$ (pg/mL)	estimated
27	1.8	$10^{-3} - 10^{3}$	$[IL\text{-}4]$ (pg/mL)	estimated

28	2.4×10^{-2}	$10^{-3} - 10^{3}$	$[IL\text{-}4]$ (pg/mL)	estimated
29	1.5×10^{2}	$10^{2} - 10^{4}$	$[IL\text{-}6]$ (pg/mL)	estimated
30	5.0×10^{-2}	$10^{-3} - 10^{2}$	$[IL\text{-}12]$ (pg/mL)	estimated
31	2.0	$10^{-3} - 10^{2}$	$[IL\text{-}12]$ (pg/mL)	estimated
32	2.0×10^{-1}	$10^{-3} - 10^{2}$	$[IFN\gamma]$ (pg/mL)	estimated
33	3.3	$10^{-1} - 10^{3}$	$[IL\text{-}4]$ (pg/mL)	estimated
34	1.6×10^{2}	$10^{2} - 10^{4}$	$[IL\text{-}6]$ (pg/mL)	estimated
35	7.7×10^{-2}	$10^{-6} - 10^{1}$	(pg/mL)/T-cell	estimated from [16]
36	1.0×10^{1}	$10^{-6} - 10^{1}$	(pg/mL)/cell	estimated from [16]
37	9.1×10^{-4}	$10^{-10} - 10^{-1}$	(pg/mL)/cancer cell	estimated from [16]
38	9.8×10^{-4}	$1.4 \times 10^{-4} - 1.4 \times 10^{-1}$	$\big((\text{pg/mL}) \cdot \text{day}\big)^{-1}$	estimated
39	2.5	$1.4 - 1.4 \times 10^{2}$	day^{-1}	estimated
40	1.5×10^{-3}	$1.4 \times 10^{-3} - 1.4 \times 10^{-1}$	$\big((\text{pg/mL}) \cdot \text{day}\big)^{-1}$	estimated
41	4.5×10^{-3}	$10^{-3} - 10^{5}$	$[PD\text{-}1 : PD\text{-}L1]$ (pg/mL)	estimated
42	9.6×10^{-1}	$10^{-3} - 10^{5}$	$[PD\text{-}1 : PD\text{-}L1]$ (pg/mL)	estimated
43	2.2	$10^{-3} - 10^{5}$	$[PD\text{-}1 : PD\text{-}L1]$ (pg/mL)	estimated
44	2.4×10^{-2}	see text	$\frac{\text{pg/mL}}{Th_2 \text{ cell} \cdot \text{day}}$	–
45	3.1×10^{-3}	see text	$\frac{\text{pg/mL}}{Th_2 \text{ cell} \cdot \text{day}}$	–
46	3.9×10^{1}	see text	$\frac{\text{pg/mL}}{T_c \text{ cell} \cdot \text{day}}$	–
47	3.8×10^{-5}	see text	$\frac{\text{pg/mL}}{T_c \text{ cell} \cdot \text{day}}$	–
**Protein**	**Nominal value**	**Range**	**Units**	**Reference**
IFNγ	0.38	0.18 – 482.31	pg/mL	patient data
IL-12	1.76	1.82 – 11.44	pg/mL	patient data
IL-6	7626.67	149.15 – 35884.0	pg/mL	patient data
IL-4	0.62	0.10 – 61.37	pg/mL	patient data
**Cell fraction**	**Nominal value**	**Range**	**Units**	**Reference**
Cancer fraction	0.30	0.1 – 0.9*	–	estimated
TN8 fraction	0.65	0.21–0.97	–	patient data
Tc fraction	0.10	0.0–0.59	–	patient data
CD4+ fraction	0.25	0.01–0.69**	–	patient data
Th1 fraction	2.0×10^{-3}	0 – 0.99	–	estimated
Th2 fraction	7.9×10^{-3}	0 – 0.99***	–	estimated

*The tumor is assumed to consist of a population of cancer cells and a population of immune cells. Thus with the nominal values given in Supplementary Table 3, 81% of the tumor is cancer cells and the remaining 19% is the total immune cell population.
**The total immune cell population consists of naive CD8+ T-cells (T_{N8}), CD8+ cytotoxic T-cells (T_c), and a population of CD4+ cells, thus we always impose the constraint (TN8 fraction + Tc fraction + CD4+ fraction) = 1.
***The CD4+ fraction is further subdivided into naive helper CD4+ T-cells (T_{N4}), type 1 helper T-

cells (Th_1) and type 2 helper T-cells (Th_2), thus we always impose the constraint (Th1 fraction + Th2 fraction + TN4 fraction) = 1.

C Mathematical modeling of recombinant IL-12 and IL-6 inhibition

Administration of recombinant IL-12 is assumed to increase the total IL-12 concentration in the system, thus directly affecting the time evolution of the populations of naive helper (CD4+) T-cells, type 1 helper T-cells, and CD8+ cytotoxic T-cells, as described below. All other equations remain identical to those presented in Appendix A unless otherwise specified.

1. The initial recombinant IL-12 dose is assumed to undergo natural decay, thus the time evolution of recombinant IL-12, $[R\text{-}IL12]$, is described by:

$$\frac{d[R\text{-}IL12]}{dt} = -\delta_{R\text{-}IL12}[R\text{-}IL12], \tag{18}$$

where the decay rate is taken to correspond to a drug half-life of 30 hours [17].

2. We then define the total IL-12 concentration, $[T\text{-}IL12]$, as:

$$[T\text{-}IL12] = [R\text{-}IL12] + [IL\text{-}12], \tag{19}$$

where the time evolution of $[IL\text{-}12]$ is given in Equation 10 of Appendix A.

Then all instances of IL-12 concentration in the equations for the naive helper (CD4+) T-cells, type 1 helper T-cells, and CD8+ cytotoxic T-cells are replaced by the total IL-12 concentration, giving:

3. Time evolution of naive helper (CD4+) T-cell population:

$$\begin{aligned}\frac{dT_{N4}}{dt} &= n_4 T_{N4} - \left(d_{1\text{-}12} T_{N4} \frac{[T\text{-}IL12]}{q_{dIL12} + [T\text{-}IL12]} + d_{1\text{-}IFN} T_{N4} \frac{[IFN\gamma]}{q_{IFN\text{-}1} + [IFN\gamma]} \right) \left(\frac{s_1}{s_1 + [PD\text{-}1 : PD\text{-}L1]} \right) \\ &\quad - \left(d_2 T_{N4} \frac{[IL\text{-}4]}{q_{dIL4} + [IL\text{-}4]} \right) \left(\frac{s_2}{s_2 + [PD\text{-}1 : PD\text{-}L1]} \right)\end{aligned} \tag{20}$$

4. Time evolution of type 1 helper T-cell population:

$$\frac{dTh_1}{dt} = n_1 Th_1 + \left(d_{1\text{-}12} T_{N4} \frac{[T\text{-}IL12]}{q_{dIL12} + [T\text{-}IL12]} + d_{1\text{-}IFN} T_{N4} \frac{[IFN\gamma]}{q_{IFN\text{-}1} + [IFN\gamma]} \right) \left(\frac{s_1}{s_1 + [PD\text{-}1 : PD\text{-}L1]} \right) \tag{21}$$

5. Time evolution of cytotoxic (CD8+) T-cell population:

$$\frac{dT_c}{dt} = n_c T_c + g_{c-12} T_c \frac{[T\text{-}IL12]}{q_{gIL12} + [T\text{-}IL12]} + d_c T_{N8} \left(\frac{Th_1}{q_1 + Th_1} \right) \left(\frac{s_c}{s_c + [PD\text{-}1 : PD\text{-}L1]} \right). \tag{22}$$

The mathematical modeling of the IL-6 inhibitor, siltuximab, is much simpler as described below.

1. Siltuximab, $[S]$, is assumed to undergo natural decay, thus the time evolution of the drug is:

$$\frac{d[S]}{dt} = -\delta_S[S], \tag{23}$$

where the decay rate is taken to correspond to a drug half-life of 20.6 days [18].

2. The effect of siltuximab is then modeled to decrease the overall production rate of IL-6, whose time evolution is now given by:

$$\frac{d[IL\text{-}6]}{dt} = \left(p_{2\text{-}6} Th_2 + p_{Can\text{-}6} C\right)\left(1 - IL6_{\text{rate}}[S]\right) - \delta_{IL6}[IL\text{-}6]. \tag{24}$$

In this way, the siltuximab dose is irrelevant, but the rate of IL-6 inhibition still depends on the drug concentration via the term $IL6_{\text{rate}}[S]$, which decays over time. Therefore, the maximal rate of IL-6 inhibition is controlled by the value of $IL6_{\text{rate}}$, which is typically taken to be 0.01 (i.e. 1% inhibition at maximal siltuximab concentration) in numerical simulations. The effects of IL-6 inhibition on the rest of the system then occur indirectly as a result of the reduced IL-6 concentration.

The results of simulating IL-6 inhibition combined with the nivolumab treatment protocol are depicted in Supplementary Figure 2. In Supplementary Figure 2(a), we show the percentage of 1,000 non-responders to anti-PD-1 immunotherapy that were converted to responders by administering a single dose of IL-6 inhibitor at different times before the start of nivolumab treatment. In Supplementary Figure 2(b), we depict the corresponding changes to the tumor micro-environment.

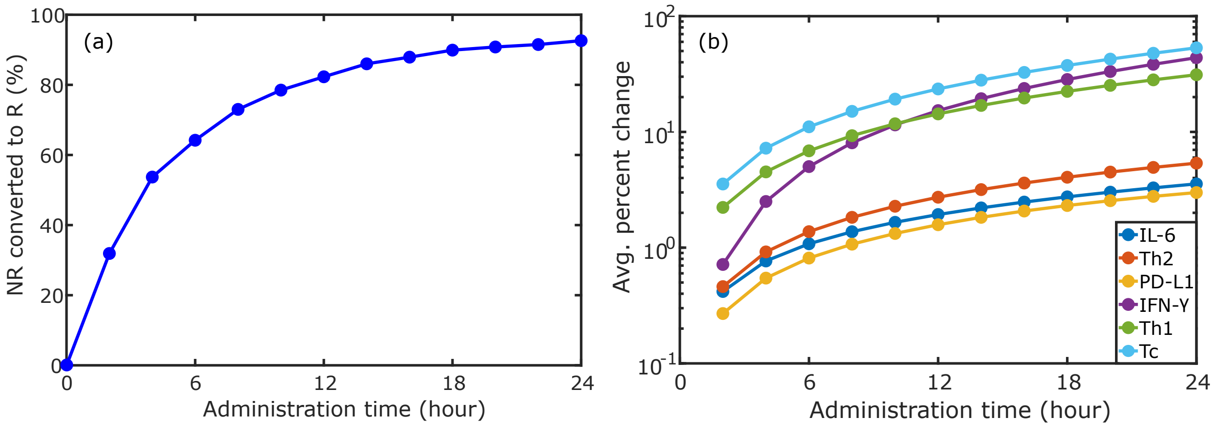

Figure 2: Simulation results of IL-6 inhibitor combined with the nivolumab treatment protocol for non-responders to anti-PD1 immunotherapy. (a) Percent of patients converted from non-responders to responders at a fixed single dose of IL-6 inhibitor (maximum 1% reduction in IL-6 production rate) administered at different times *before* nivolumab treatment protocol. (b) Corresponding absolute value of average percent change of cytokine and T-cell populations. Average was taken over 1,000 patients, calculated between the time of IL-6 inhibitor administration and just prior to nivolumab treatment protocol. For IL-6, Th-2 and PD-L1, average percent change is negative, corresponding to inhibition; for all other quantities, average percent change is positive. For both (a) and (b), the 1,000 patients were chosen from the non-responder group by selecting patients with the smallest tumor size after the nivolumab treatment protocol (note that the tumor size was larger than before nivolumab treatment per our definition of 'non-responder'). Administration time is the number of hours IL-6 inhibitor was given prior to the start of the nivolumab treatment protocol.

D Neural network performance metrics and loss functions

In Supplementary Table 4, we give the definition of the performance metrics used in this work for binary classification. In this work, responders were taken to be class 1 (positive) and non-responders to be class 0 (negative).

During training of the non-linear regression systems biology informed neural network (NR-SBINN), the loss function was taken to be the mean-squared error, defined by:

$$\text{MSE} = \frac{1}{N}\sum_{i=1}^{N}\left(y_i - \hat{y}_i\right)^2,$$

where the sum is taken over all samples N, y_i is the true value of sample i, and $\hat{y}_i$ is the predicted value of sample i. A representative learning curve for the NR-SBINN using this loss function is presented in Supplementary Figure 3.

For training the classification systems biology informed neural network (C-SBINN), the loss function was taken to be binary cross entropy, which is defined by:

$$\text{BCE} = -\frac{1}{N}\sum_{i=1}^{N}\left(y_i \log\left(p(y_i)\right) + (1 - y_i)\log\left(1 - p(y_i)\right)\right),$$

Table 4: Performance metrics used for patient response classification.

Term	Definition	Description
True positive (TP)		An outcome where the model correctly predicts the positive class
True negative (TN)		An outcome where the model correctly predicts the negative class
False positive (FP)		An outcome where the model incorrectly predicts the positive class
False negative (FN)		An outcome where the model incorrectly predicts the negative class
True positive rate (TPR)	$\frac{\text{TP}}{\text{TP+FN}} = 1 - \text{FNR}$	Also called the sensitivity.
True negative rate (TNR)	$\frac{\text{TN}}{\text{TN+FP}} = 1 - \text{FPR}$	Also called the specificity.
False positive rate (FPR)	$\frac{\text{FP}}{\text{TN+FP}} = 1 - \text{TNR}$	
False negative rate (FNR)	$\frac{\text{FN}}{\text{TP+FN}} = 1 - \text{TPR}$	
accuracy	$\frac{\text{TP+TN}}{\text{TP+TN+FP+FN}}$	
precision	$\frac{\text{TP}}{\text{TP+FP}}$	
recall		Defined as the TPR.
geometric mean (G-mean)	$\sqrt{\text{TPR} \cdot \text{TNR}}.$	Values fall in the range $[0, 1]$, with 1 corresponding to perfect classification [19].
F1-score	$2\frac{\text{precision}\cdot\text{recall}}{\text{precision}+\text{recall}}$	Harmonic mean of precision and recall. Values fall in the range $[0, 1]$, with 1 corresponding to perfect classification [20, 21].
Matthew correlation coefficient (MCC)	$\frac{\text{TP}\cdot\text{TN}-\text{FP}\cdot\text{FN}}{\sqrt{(\text{TP+FP})(\text{TP+FN})(\text{TN+FP})(\text{TN+FN})}}$	Values fall in the range $[-1, 1]$, with 1 corresponding to perfect classification, 0 corresponding to no better than random prediction, and -1 indicating complete disagreement between prediction and observation [22].
Cohen kappa score (CKS)	$\frac{2(\text{TP}\cdot\text{TN}-\text{FP}\cdot\text{FN})}{(\text{TP+FP})(\text{TP+FN})+(\text{TN+FP})(\text{TN+FN})}$	A CKS value of 1 corresponds to perfect classification, 0 corresponds to no better than random prediction, and < 0 indicates worse than random agreement [23, 24].

where the sum is taken over all samples N, log is the natural logarithm, y_i is the class label (0 or 1) of sample i, and $p(y_i)$ the predicted probability of sample i belonging to class 1. Representative learning curves for the C-SBINN using this loss function are depicted in Supplementary Figure 4.

E Hyperparameter tuning

A simple grid search was used along with Tensorboard [25] to determine the optimal network hyperparameters for the non-linear regression systems biology informed neural network (NR-SBINN). During this process, the NR-SBINN was trained and tested on a subset of the simulated clinical data described in Section 4.4.2 that was reserved for hyperparameter tuning. The number of hidden layers, number of hidden nodes in each layer, activation function in the hidden layers, learning rate and learning rate decay value, layer weight initializers, and batch size were taken as optimization parameters. The optimal NR-SBINN for an input layer with four inputs (corresponding to the IFNγ levels at $t = 0$, 24, 48, and 72 hr) was found to consist of the following network architecture: two hidden layers with, respectively, 24 and 8 neurons each; Leaky ReLU activation functions in the hidden layers with $\alpha = 0.001$; learning rate of 0.001 and decay value 0.001; Keras He normal initializer with a seed value of zero; batch size of 128 samples. The network was fully connected, and the output layer consisted of a single output neuron, with no activation function since the network was performing nonlinear regression. Using this network archi-

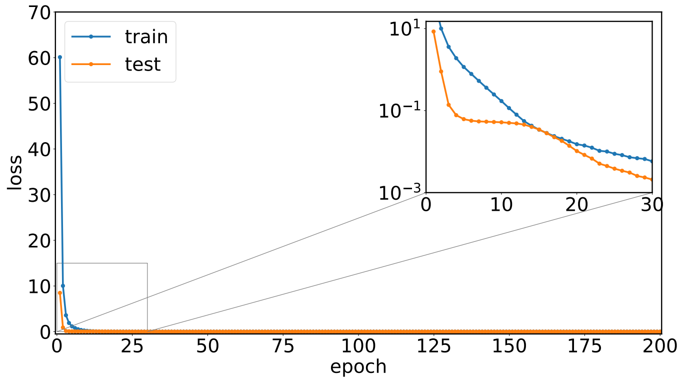

Figure 3: Calculated loss function, taken to be the mean-squared error, during training and validation of the NR-SBINN to predict the value of parameter 14 from IFNγ expression levels.

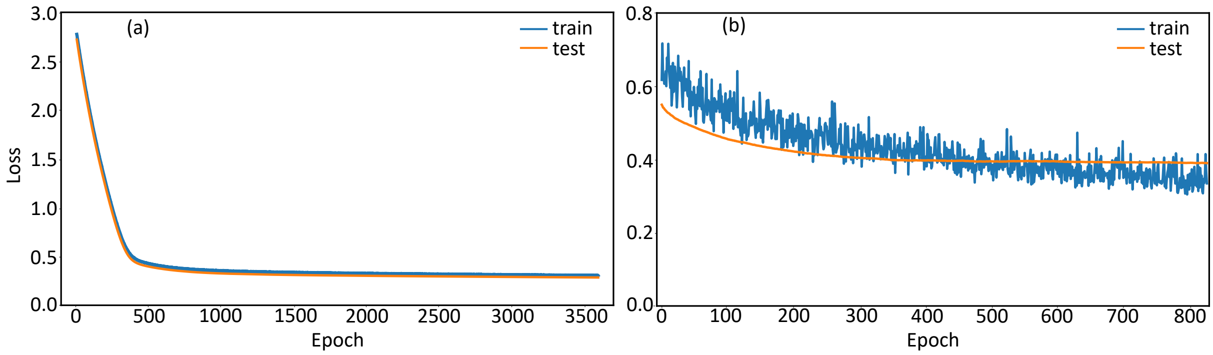

Figure 4: Training loss, calculated as the binary cross entropy, for the C-SBINN for (a) imbalanced simulated clinical data set and (b) ex-vivo patient data set with transfer learning.

tecture and learning protocol, the NR-SBINN also achieved a high accuracy on the remaining simulated patient data, converging after a small number of training epochs, see Supplementary Figure 3.

A more sophisticated approach, Bayesian optimization, was implemented for the classification systems biology informed neural networks (C-SBINN) using the Python scikit-optimize library, specifically the gp_minimize optimizer with the negative expected improvement (EI) function chosen for minimizing over the Gaussian prior. For all classification neural networks, the activation functions in the hidden layers were taken to be LeakyReLU, and the output layer consisted of a single neuron with sigmoid activation function. The He normal initializer was used to initialize the network weights in the layers with LeakyReLU activation function (seed value of zero), and the Glorot normal initializer was used to initialize the weights in the output layer (with seed value zero). (These choices, along with the default network hyperparameters for the Bayesian optimization, were identified from a simple preliminary grid search). In the case of transfer learning, Bayesian optimization was used to determine the optimal learning rate, learning rate decay value, number of hidden layers, number of nodes in each hidden layer, α parameter for the LeakyReLU activation function, dropout between network layers, batch size for the simulated clinical data, batch size for the ex-vivo patient data, number of training epochs for the simulated clinical data, and number of training epochs for the ex-vivo patient data. In the case of no transfer learning, Bayesian optimization was used to determine the optimal learning rate, learning rate decay value, number of hidden layers, number of nodes in each hidden layer, α parameter for the LeakyReLU activation function, dropout between network layers, batch size for the ex-vivo patient data, and number of training epochs for the ex-vivo patient data. The search range for each parameter is presented in Supplementary Table 5.

Table 5: Bayesian optimization search ranges for the classification neural network architecture and learning hyperparameters. LR- learning rate, LRD- learning rate decay value, HL- number of hidden layers, NHL_1- number of nodes in the first hidden layer, NHL_2- number of nodes in the remaining hidden layers, α- α parameter for the LeakyReLU activation function, D- dropout between network layers, B_S- batch size for the simulated clinical data, B_E- batch size for the ex-vivo patient data, T_S- number of training epochs for the simulated clinical data, T_E- number of training epochs for the ex-vivo patient data.

LR, LRD	HL	NHL_1, NHL_2	α	D	B_S	B_E	T_S	T_E
$[10^{-6}, 0.1]$	$[1, 4]$	$[5, 256]$	$[10^{-4}, 0.1]$	$[0, 0.5]$	$[1024, 4096]$	$[1, 16]$	$[100, 4500]$	$[50, 1500]$

Briefly, for all sets of input features/learning approaches described in Section 4.4.3 of the manuscript, 20 optimization steps were performed, and five-fold cross-validation was performed at each optimization step. Each cross-validation fold involved randomly splitting the data into training and testing set sets as described in Section 4.4.3 of the manuscript. For the transfer learning approaches, the network was pre-trained on the imbalanced simulated clinical trial data and then re-trained on the ex-vivo training data set, and the fully trained model was applied to the ex-vivo testing set. This was repeated for all five-folds, then the average ROC area under the curve was calculated for the testing set and taken as the fitness value. (Note that we also performed this procedure with the average PRC area under the curve and the average MCC on the testing set taken as the fitness value. Using either the area under the ROC or PRC curve led to the most stable networks with the ability to generalize to unseen data). After all optimization steps, the set of network hyperparameters with the highest average fitness value was taken as the optimal neural network. For the cases with no transfer learning, the procedure was identical except there was no pre-training on simulated patient data. As indicated above, this procedure was performed separately for the tumor micro-environment features, response features, patient-selected response features, and all ex-vivo measurements used as inputs, leading to different optimal neural networks for these different cases. The optimal networks determined for each set of features and learning approach, which were used throughout the work, are described in Supplementary Table 6. During training, the loss function (binary cross-entropy) was monitored to check for overfitting, see for example, Supplementary Figure 4.

To validate the results of the hyperparameter tuning and investigate whether the above procedure led to over-optimistic classification results, we also separately performed nested cross-validation, which is significantly more computationally expensive. This procedure enabled the determination of how well the learning strategies generalized to completely unseen data, validating the results presented in Section 2.3 of the manuscript. This procedure involved setting up five outer cross-validation folds and five inner cross-validation folds. For each outer cross-validation fold, the ex-vivo data was randomly split into an

Table 6: Optimal neural network architectures and learning parameters for different input features and learning approaches, determined by Bayesian optimization with the area under the ROC curve taken as the fitness value. Abbreviations are identical to Supplementary Table 5.

**Tumor micro-environment features, with transfer learning**										
LR	LRD	HL	NHL_1	NHL_2	α	D	B_S	B_E	T_S	T_E
5.5×10^{-6}	1.4×10^{-6}	2	21	126	0.017	0.019	3200	5	3588	250
**Tumor micro-environment features, without transfer learning**										
LR	LRD	HL	NHL_1	NHL_2	α	D	B_S	B_E	T_S	T_E
2.5×10^{-6}	1.0×10^{-6}	4	156	138	0.042	0.38	-	13	-	1500
**Response features selected from simulated data, with transfer learning**										
LR	LRD	HL	NHL_1	NHL_2	α	D	B_S	B_E	T_S	T_E
6.1×10^{-6}	8.2×10^{-5}	3	80	256	9.0×10^{-4}	0.069	3734	1	2997	830
**Response features selected from simulated data, without transfer learning**										
LR	LRD	HL	NHL_1	NHL_2	α	D	B_S	B_E	T_S	T_E
1.2×10^{-3}	0.1	3	112	55	0.027	0.011	-	10	-	1374
**Response features selected from ex-vivo data, without transfer learning**										
LR	LRD	HL	NHL_1	NHL_2	α	D	B_S	B_E	T_S	T_E
1.6×10^{-5}	2.6×10^{-5}	1	248	-	2.5×10^{-4}	0.056	-	10	-	179
**All ex-vivo experimental measurements, without transfer learning**										
LR	LRD	HL	NHL_1	NHL_2	α	D	B_S	B_E	T_S	T_E
1.4×10^{-3}	5.6×10^{-2}	2	90	64	0.081	0.43	-	12	-	376

outer training set and an outer testing set that approximately preserved the class imbalance of the whole ex-vivo data set (32 training samples, 5 testing samples). Then 20 steps for the Bayesian optimization algorithm were performed. For each optimization step, the five-fold inner cross-validation was performed. During each inner cross-validation fold, the outer ex-vivo training set was randomly split into an inner training set and an inner testing set that approximately preserved the class imbalance of the whole ex-vivo data set (27 training samples, 5 testing samples). The Python scikit-learn StandardScalar function was then fit to the inner ex-vivo training set and subsequently applied to the remaining ex-vivo data (i.e. the inner ex-vivo testing set and the outer ex-vivo testing set). The candidate neural network was then trained on the simulated clinical data followed by re-training on the inner ex-vivo training set (in the case of transfer learning), or just on the inner ex-vivo training set (in the case of no transfer learning). Then the trained network was applied to the inner ex-vivo testing set and the fitness value was calculated for that inner cross-validation fold. This was repeated for all inner cross-validation folds and the average fitness value over all inner folds was used to estimate the performance of the candidate neural network.

After all 20 Bayesian optimization steps were performed (each with five-fold inner cross-validation as explained above), the neural network with the highest average fitness value was selected. This neural network was then trained on the simulated clinical data followed by re-training on the entire outer ex-vivo training set (in the case of transfer learning), or just the outer ex-vivo training set (in the case of no transfer learning). The trained neural network was then applied to the outer ex-vivo testing set. Since this testing set was withheld during the model selection process, the results obtained from the outer ex-vivo testing set indicate how well the modeling approach generalizes to unseen data. The entire process was repeated for each of the five outer cross-validation folds, and the average of the performance metrics on the outer ex-vivo testing sets were calculated. We present the results in Supplementary Table 7 for the tumor micro-environment features with and without transfer learning. Importantly, we see, based on the ROC (AUC) and PRC (AUC), that the modeling approach does indeed generalize well to unseen data in

the case of transfer learning, which validates the results in Table 3 of the manuscript. Furthermore, we see that without transfer learning, the results in Supplementary Table 7 are quite a bit lower than those reported in Table 3. This suggests that the results in Table 3 may be overestimating the ability of the classification neural network to generalize to unseen data when it is trained only on the small ex-vivo data set. This further highlights the importance of a transfer learning approach for small data sets.

Table 7: Nested cross-validation results obtained with and without transfer learning, using the tumor micro-environment features as inputs to the classification neural network. ROC (AUC) is the area under the receiving-operator characteristic curve, PRC (AUC) is the area under the precision-recall curve, CKS is the Cohen kappa score, and MCC is the Matthew correlation coefficient. The nested cross-validation consisted of five folds in the outer cross-validation loop, 20 Bayesian optimization steps for each outer fold, and five folds in the inner cross-validation loop for each Bayesian optimization step. Average value of performance metrics on the outer testing sets are shown. The average ROC (AUC) on the inner testing sets was taken as the fitness value for model selection (see text for details). Similar results were obtained by taking the average PRC (AUC) as the fitness value.

**Transfer learning**				
ROC (AUC)	PRC (AUC)	MCC	CKS	Optimal threshold
0.85	0.68	0.52	0.51	0.07
**No transfer learning**				
ROC (AUC)	PRC (AUC)	MCC	CKS	Optimal threshold
0.55	0.26	0.03	0.05	0.62

To calculate the MCC and CKS, the classification threshold must be optimized for imbalanced data sets, and there are conceivably many ways this can be done during the nested cross-validation process. We found that the best approach to determine an optimal classification threshold for each outer cross-validation fold was as follows. After the candidate neural network was trained during the inner cross-validation fold as explained above, the classification threshold that led to maximum MCC on both the inner ex-vivo training and inner ex-vivo testing sets was recorded. The average classification threshold over the five inner-cross validation folds was then recorded and associated with the candidate neural network. In the outer cross-validation fold, the threshold associated with the optimal neural network was used for calculating the MCC and CKS on the outer ex-vivo testing data after the neural network was trained on the outer ex-vivo training data. In this way, only the outer ex-vivo training data was used to determine the classification threshold for each outer cross-validation step. The average MCC and CKS, computed over all outer cross-validation folds, are presented in Supplementary Table 7 along with the other performance metrics for the cases of transfer learning and no transfer learning. Importantly, these results agree with the MCC (all) and CKS (all) results in Table 3 of the manuscript, which were taken as an estimate for how well the learning approach would generalize to unseen data. This agreement further validates the notion that transfer learning helps the machine learning algorithm generalize to unseen data and is therefore crucial when dealing with small clinical data sets.

F Ex vivo patient labels

The C-SBINN was used to predict whether a patient is a probable responder or probable non-responder to anti-PD-1 immunotherapy using measurements from ex-vivo experiments that incorporate fragments from the patient's tumor biopsy. While the trained C-SBINN can be applied to new data sets to predict whether patients are likely to respond favorably to treatment, the training process requires the ex-vivo data to be labelled according to the patient clinical response. In an ideal setting, such information would be readily available; however, the authors did not have access to such labels in this work. In the absence of matched-patient clinical information, we sought to develop an independent statistical approach to label the ex-vivo data. To ensure independence, the approach we developed used experimental measurements of granzyme and perforin expression and the CD8+:CD4+ T-cell ratio, all of which were not directly simulated in the systems biology model or used as inputs into the C-SBINN. Granzyme and perforin

expression are released in the granules of cytotoxic T-cells and natural killer cells to induce apoptosis, thus their expression patterns are expected to correlate in some capacity with the clinical treatment response. In addition, the CD8+:CD4+ T-cell ratio has been shown to have prognostic value in patients with cancer and auto-immune diseases [26, 27].

Table 8: Metrics used for k-means clustering (with $k = 2$) to label patients as 'responders' or 'non-responders' to anti-PD-1 immunotherapy. Data was standardized for the metrics indicated in the table. Average expression levels refer to the calculated average of three measurements over the 72-hour experiment window.

Metric	Description	Standardized (yes/no)	Average patient response rate (%)	Number of responders
1	Ratio of treatment to control measurement for: CD8+:CD4+ ratio at 72 hr, average granzyme level, average perforin level, peak granzyme level, peak perforin level	no	36.6	12
2	Same as metric 1	yes	49.2	22
3	Average control granzyme level, average control perforin level, average treatment granzyme level, average treatment perforin level, peak control granzyme level, peak control perforin level, peak treatment granzyme level, peak treatment perforin level	no	13.6	8
4	Same as metric 3	yes	14.3	9
5	Same as metric 3 plus control CD8+:CD4+ ratio at 72 hr and treatment CD8+:CD4+ ratio at 72 hr	yes	24.7	9
6	All ratios and measurements in metrics 1-5	yes	23.5	12
7	Relative change of treatment to control measurement for: granzyme level and perforin level (both at 72 hr)	no	20.1	10
8	Treatment granzyme level, treatment perforin level, and treatment CD8+:CD4+ ratio (all at 72 hr)	yes	17.6	10
9	Ratio of treatment to control measurement for: granzyme level, perforin level, and CD8+:CD4+ ratio (all at 72 hr)	yes	31.3	16

While suitable features were identified for labeling patients as 'responders' or 'non-responders' to anti-PD-1 immunotherapy, it was not clear precisely how these features should be used in practice. For example, one could take the peak measurements over the 72-hour treatment window, or the average of the measurements, or the ratio of these measurements to their control values, or another variation. To overcome this obstacle and to eliminate the possibility that bias in the chosen metric might affect the patient label, we incorporated several different metrics, which are described in Supplementary Table 8, into an unsupervised machine learning technique called k-means clustering [28]. The k-means clustering algorithm is sensitive to the scale (variance) in the data. Therefore we considered metrics for which the data was standardized as well as metrics for which the data was not scaled. Additionally, the initialization of the k-means clustering algorithm is based upon the generation of random numbers, thus some patients were seen to toggle between the 'responder' and 'non-responder' classes when the clustering was repeated.

To overcome these limitations and to develop a more robust statistical approach, the k-means clustering was performed 100 times for each metric listed in Supplementary Table 8.

The procedure for classifying patients using each metric was as follows. For each patient, the assigned class was recorded for each run (it was assumed that the response cluster corresponded to higher average granzyme and perforin levels than the non-response cluster). After 100 runs, the response rate for each patient was calculated by tallying the number of times they were assigned to the 'responder' class. Next it was necessary to determine if a patient was a 'probable responder' by comparing their response rate for the metric with an arbitrary threshold value. At first, it might seem intuitive to set the threshold value to be 50%, i.e. if a patient falls into the response cluster more than 50% of the time, they should be a 'probable responder'. However, as we show in Supplementary Table 8, the average patient response rate changes significantly between metrics, varying between 13.6 and 49.2%. In an attempt to reduce the ambiguity in threshold response, we chose the threshold for each metric to be the average patient response rate for that metric. Therefore, patients whose response rate was above the metric-specific threshold were considered 'probable responders' for this metric, and those below the threshold were considered 'probable non-responders'.

This process was repeated for each of the metrics listed in Supplementary Table 8. For most metrics, the number of predicted responders varied between 8 and 12 (out of 37) patients, though we see from Supplementary Table 8 that metrics two and nine were outliers. To finally assign a class label to a patient, we looked at how the patient was clustered for each of the nine metrics. Patients who were deemed to be 'probable responders' for five or more metrics were labeled as 'responders' and those who were deemed 'probable responders' for four or less metrics were labeled as 'non-responders'. Using this approach, it was determined that eight of 37 patients (just under 22 %) were 'responders'. We note that lowering the cutoff to four instead of five out of nine metrics results in $13/37 = 35\%$ of patients labeled as 'responders'. In the training of the C-SBINN, we used the cutoff of 5/9 since this response rate was more consistent with previously reported patient response rates for anti-PD1 immunotherapy [29]. The granzyme and perforin expression under control and treatment conditions for the two classes are depicted in Supplementary Figure 5 and illustrate the statistically significant differences between the groups under both conditions.

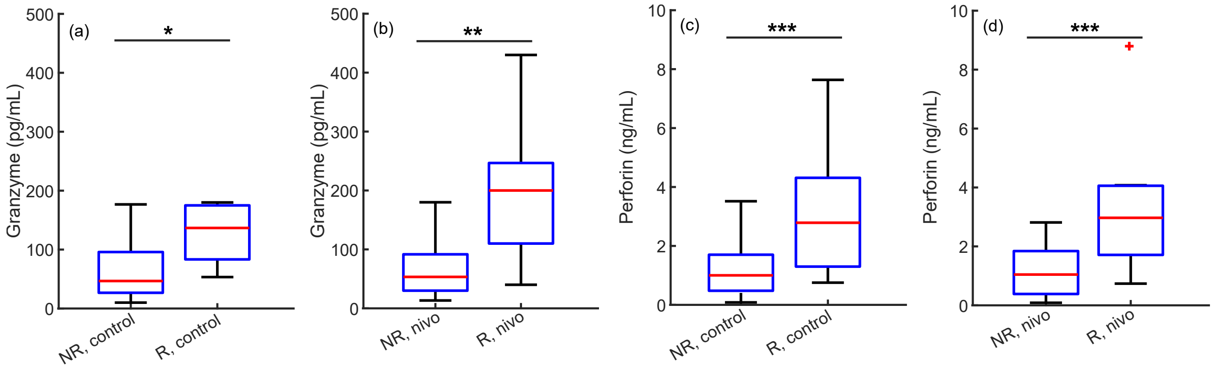

Figure 5: Average ex-vivo granzyme and perforin expression for patients classified as 'responders' (R) and 'non-responders' (NR) using k-means clustering under vehicle control and nivolumab (nivo) treatment conditions. Boxplots depict the statistically significant differences between the two response groups: *$p<0.02$, **$p<0.001$, ***$p<0.01$ by Mann-Whitney U test.

One drawback to using high-dimensional metrics to cluster the patient data is that it is difficult to visualize the clusters in greater than three dimensions. To overcome this challenge, we used the t-distributed stochastic neighbor embedding (tSNE) algorithm [30], implemented in Matlab, for visualization. The tSNE algorithm is a nonlinear dimensionality reduction technique which is well-suited for embedding high-dimensional data for visualization in a low-dimensional space (of two or three dimensions). We considered four different distance measures (Mahalanobis, Cosine, Chebychev, and Euclidean), and for most of the metrics in Supplementary Table 8 the Euclidean distance led to the best separation between clusters in both two and three dimensions. In Supplementary Figure 6, we present the cluster results embedded in both two and three dimensions using the tSNE algorithm with Euclidean distance measure for k-means clustering using metric three. As shown in the figure, metric three resulted in eight predicted responders.

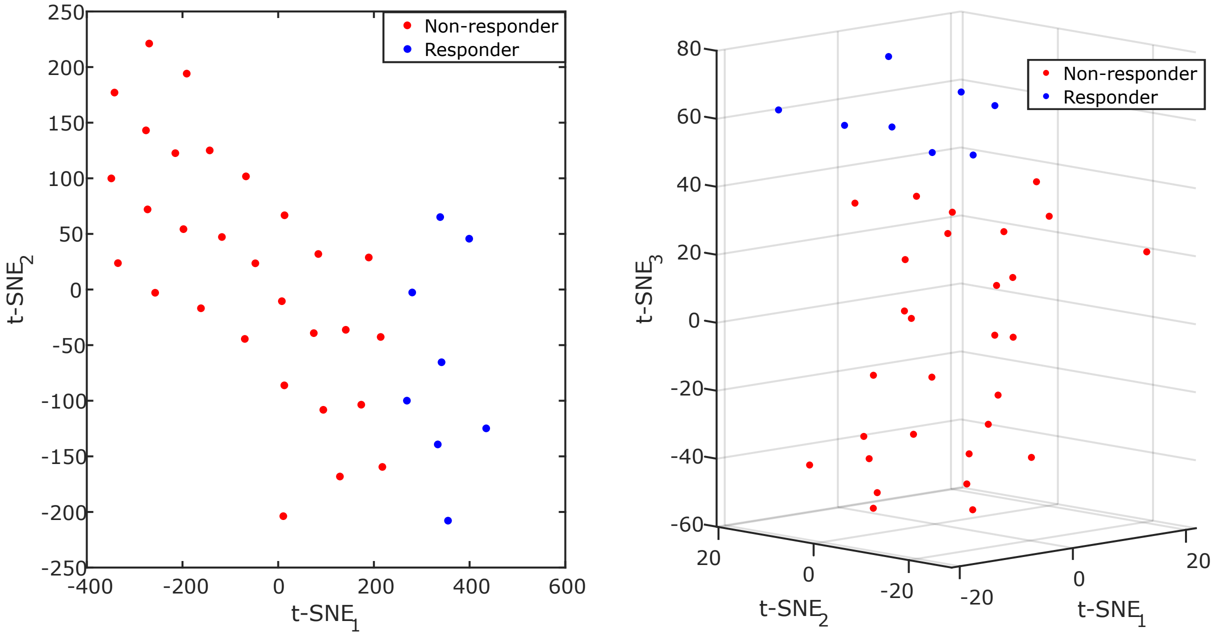

Figure 6: Cluster results embedded in two and three dimensions using the tSNE algorithm with Euclidean distance measure. The clusters were determined using the k-means clustering algorithm with metric three in Supplementary Table 8, which led to a predicted eight responders. Axes labels indicate the tSNE dimensions.

Supplementary References

[1] Andrew Yates, Claudia Bergmann, J Leo Van Hemmen, Jaroslav Stark, and Robin Callard. Cytokine-modulated regulation of helper t cell populations. *Journal of theoretical biology*, 206(4):539–560, 2000.

[2] Sean Diehl and Mercedes Rincón. The two faces of il-6 on th1/th2 differentiation. *Molecular immunology*, 39(9):531–536, 2002.

[3] Gordon J Freeman, E John Wherry, Rafi Ahmed, and Arlene H Sharpe. Reinvigorating exhausted hiv-specific t cells via pd-1–pd-1 ligand blockade. *Journal of Experimental Medicine*, 203(10):2223–2227, 2006.

[4] Taku Okazaki and Tasuku Honjo. The pd-1–pd-l pathway in immunological tolerance. *Trends in immunology*, 27(4):195–201, 2006.

[5] Mario Sznol and Lieping Chen. Antagonist antibodies to pd-1 and b7-h1 (pd-l1) in the treatment of advanced human cancer—response. *Clinical Cancer Research*, 19(19):5542–5542, 2013.

[6] Michael A Fishman and Alan S Perelson. Th1/th2 cross regulation. *Journal of theoretical biology*, 170(1):25–56, 1994.

[7] Shimon Sakaguchi. Regulatory t cells: key controllers of immunologic self-tolerance. *Cell*, 101(5):455–458, 2000.

[8] John Paul Ridge, Francesca Di Rosa, and Polly Matzinger. A conditioned dendritic cell can be a temporal bridge between a cd4+ t-helper and a t-killer cell. *Nature*, 393(6684):474, 1998.

[9] Witold Lasek, Radosław Zagożdżon, and Marek Jakobisiak. Interleukin 12: still a promising candidate for tumor immunotherapy? *Cancer Immunology, Immunotherapy*, 63(5):419–435, 2014.

[10] Joseph A Trapani and Mark J Smyth. Functional significance of the perforin/granzyme cell death pathway. *Nature Reviews Immunology*, 2(10):735, 2002.

[11] Michael A Fishman and Alan S Perelson. Th1/th2 differentiation and cross-regulation. *Bulletin of mathematical biology*, 61(3):403–436, 1999.

[12] Sergio Romagnani. The th1/th2 paradigm. *Immunology today*, 18(6):263–266, 1997.

[13] Mercedes Rincón, Juan Anguita, Tetsuo Nakamura, Erol Fikrig, and Richard A Flavell. Interleukin (il)-6 directs the differentiation of il-4–producing cd4+ t cells. *Journal of Experimental Medicine*, 185(3):461–470, 1997.

[14] Steven E Macatonia, Nancy A Hosken, Mark Litton, Paulo Vieira, Chyi-Song Hsieh, Janice A Culpepper, Maria Wysocka, Giorgio Trinchieri, Kenneth M Murphy, and Anne O'Garra. Dendritic cells produce il-12 and direct the development of th1 cells from naive cd4+ t cells. *The Journal of Immunology*, 154(10):5071–5079, 1995.

[15] Benoit F Morel, Jayant Kalagnanam, and Penelope A Morel. Mathematical modeling of th1-th2 dynamics. In *Theoretical and experimental insights into immunology*, pages 171–190. Springer, 1992.

[16] Xiulan Lai and Avner Friedman. Combination therapy of cancer with cancer vaccine and immune checkpoint inhibitors: A mathematical model. *PLoS One*, 12(5):e0178479, 2017.

[17] Vicente Carreño, Stefan Zeuzem, Uwe Hopf, Patrick Marcellin, W Graham E Cooksley, Johan Fevery, Moisés Diago, Rajender Reddy, Marion Peters, Karen Rittweger, et al. A phase i/ii study of recombinant human interleukin-12 in patients with chronic hepatitis b. *Journal of hepatology*, 32(2):317–324, 2000.

[18] Shayna Sarosiek, Ruchit Shah, and Nikhil C Munshi. Review of siltuximab in the treatment of multicentric castleman's disease. *Therapeutic advances in hematology*, 7(6):360–366, 2016.

[19] Edward B Fowlkes and Colin L Mallows. A method for comparing two hierarchical clusterings. *Journal of the American statistical association*, 78(383):553–569, 1983.

[20] David D Lewis and William A Gale. A sequential algorithm for training text classifiers. In *SIGIR'94*, pages 3–12. Springer, 1994.

[21] Qiong Gu, Li Zhu, and Zhihua Cai. Evaluation measures of the classification performance of imbalanced data sets. In *International symposium on intelligence computation and applications*, pages 461–471. Springer, 2009.

[22] Brian W Matthews. Comparison of the predicted and observed secondary structure of t4 phage lysozyme. *Biochimica et Biophysica Acta (BBA)-Protein Structure*, 405(2):442–451, 1975.

[23] Jacob Cohen. A coefficient of agreement for nominal scales. *Educational and psychological measurement*, 20(1):37–46, 1960.

[24] IH Witten, E Frank, MA Hall, and CJ Pal. Data mining fourth edition: Practical machine learning tools and techniques, 2016.

[25] Martín Abadi, Ashish Agarwal, Paul Barham, Eugene Brevdo, Zhifeng Chen, Craig Citro, Greg S. Corrado, Andy Davis, Jeffrey Dean, Matthieu Devin, Sanjay Ghemawat, Ian Goodfellow, Andrew Harp, Geoffrey Irving, Michael Isard, Yangqing Jia, Rafal Jozefowicz, Lukasz Kaiser, Manjunath Kudlur, Josh Levenberg, Dandelion Mané, Rajat Monga, Sherry Moore, Derek Murray, Chris Olah, Mike Schuster, Jonathon Shlens, Benoit Steiner, Ilya Sutskever, Kunal Talwar, Paul Tucker, Vincent Vanhoucke, Vijay Vasudevan, Fernanda Viégas, Oriol Vinyals, Pete Warden, Martin Wattenberg, Martin Wicke, Yuan Yu, and Xiaoqiang Zheng. TensorFlow: Large-scale machine learning on heterogeneous systems, 2015. Software available from tensorflow.org.

[26] Michael P Pender. Cd8+ t-cell deficiency, epstein-barr virus infection, vitamin d deficiency, and steps to autoimmunity: a unifying hypothesis. *Autoimmune diseases*, 2012, 2012.

[27] Joseph A McBride and Rob Striker. Imbalance in the game of t cells: what can the cd4/cd8 t-cell ratio tell us about hiv and health? *PLoS pathogens*, 13(11):e1006624, 2017.

[28] Stuart Lloyd. Least squares quantization in pcm. *IEEE transactions on information theory*, 28(2):129–137, 1982.

[29] Joshua M Bauml, Charu Aggarwal, and Roger B Cohen. Immunotherapy for head and neck cancer: where are we now and where are we going? *Annals of Translational Medicine*, 7(Suppl 3), 2019.

[30] Laurens van der Maaten and Geoffrey Hinton. Visualizing data using t-sne. *Journal of machine learning research*, 9(Nov):2579–2605, 2008.
