# Supplementary material for: Systems biology informed neural networks (SBINN) predict response and novel combinations for PD-1 checkpoint blockade
Source: Commun Biol. 2021 Jul 15;4:877. doi: 10.1038/s42003-021-02393-7 (PMC8282606; doi:10.1038/s42003-021-02393-7)
Supplement: Supplementary file 2 — Description of additional supplementary files [file 42003_2021_2393_MOESM2_ESM.pdf]

Description of additional supplementary files

Title: Supplementary Data 1
Description: Feature selection scores from multi-parametric sensitivity analysis (MPSA), Fisher discriminant analysis (FDA), and filter feature selection (FFS) underlying Figure 2 and Tables 1 and 2.

Title: Supplementary Data 2
Description: False positive rates and true positive rates for the receiver operator characteristics (ROC) curves in Figure 3.

Title: Supplementary Data 3
Description: Data underlying the triple combination therapy simulations (IL-6 inhibition, recombinant IL-12, nivolumab) in Figure 4.

Title: Supplementary Data 4
Description: Clinical patient data underlying the box and whisker plots in Figure 5.
