# Supplementary material for: Systems biology informed neural networks (SBINN) predict response and novel combinations for PD-1 checkpoint blockade
Source: Commun Biol. 2021 Jul 15;4:877. doi: 10.1038/s42003-021-02393-7 (PMC8282606; doi:10.1038/s42003-021-02393-7)
Supplement: Supplementary file 7 — Reporting Summary [file 42003_2021_2393_MOESM7_ESM.pdf]

nature research

Corresponding author(s): Michelle Przedborski

Last updated by author(s): Jun 18, 2021

Reporting Summary

Nature Research wishes to improve the reproducibility of the work that we publish. This form provides structure for consistency and transparency in reporting. For further information on Nature Research policies, see our Editorial Policies and the Editorial Policy Checklist.

Statistics

For all statistical analyses, confirm that the following items are present in the figure legend, table legend, main text, or Methods section.

n/a	Confirmed	
☐	☒	The exact sample size (*n*) for each experimental group/condition, given as a discrete number and unit of measurement
☐	☒	A statement on whether measurements were taken from distinct samples or whether the same sample was measured repeatedly
☐	☒	The statistical test(s) used AND whether they are one- or two-sided<br>*Only common tests should be described solely by name; describe more complex techniques in the Methods section.*
☒	☐	A description of all covariates tested
☐	☒	A description of any assumptions or corrections, such as tests of normality and adjustment for multiple comparisons
☐	☒	A full description of the statistical parameters including central tendency (e.g. means) or other basic estimates (e.g. regression coefficient) AND variation (e.g. standard deviation) or associated estimates of uncertainty (e.g. confidence intervals)
☐	☒	For null hypothesis testing, the test statistic (e.g. *F*, *t*, *r*) with confidence intervals, effect sizes, degrees of freedom and *P* value noted<br>*Give P values as exact values whenever suitable.*
☒	☐	For Bayesian analysis, information on the choice of priors and Markov chain Monte Carlo settings
☒	☐	For hierarchical and complex designs, identification of the appropriate level for tests and full reporting of outcomes
☐	☒	Estimates of effect sizes (e.g. Cohen's *d*, Pearson's *r*), indicating how they were calculated

Our web collection on statistics for biologists contains articles on many of the points above.

Software and code

Policy information about availability of computer code

Data collection	No software was used.
Data analysis	Custom code related to simulated treatment protocols for the ex vivo experiments with specified inputs and outputs, as well as comments throughout the code, can be found at https://github.com/mprzedborski/ex-vivo-PD1-blockade.

For manuscripts utilizing custom algorithms or software that are central to the research but not yet described in published literature, software must be made available to editors and reviewers. We strongly encourage code deposition in a community repository (e.g. GitHub). See the Nature Research guidelines for submitting code & software for further information.

Data

Policy information about availability of data

All manuscripts must include a data availability statement. This statement should provide the following information, where applicable:

- Accession codes, unique identifiers, or web links for publicly available datasets
- A list of figures that have associated raw data
- A description of any restrictions on data availability

Raw data used for analysis and simulations will be available upon request.

Field-specific reporting

Please select the one below that is the best fit for your research. If you are not sure, read the appropriate sections before making your selection.

☒ Life sciences ☐ Behavioural & social sciences ☐ Ecological, evolutionary & environmental sciences

For a reference copy of the document with all sections, see nature.com/documents/nr-reporting-summary-flat.pdf

Life sciences study design

All studies must disclose on these points even when the disclosure is negative.

Sample size	50 HNSCC samples were taken from consenting patients. Due to the limited availability of such primary samples no statistical methods were used to pre-determine a sample size.
Data exclusions	The final number of patient samples included in the dataset for the systems biology approach was 37. This was due to an incomplete dataset of cytokine and flow cytometry measurements from 13 patient samples.
Replication	Each tumor biopsy was fragmented into approx. 500um slices that were used for a control and a nivolumab-treated drug arm. For each drug arm three tumor slices were cultured to capture the heterogeneity inherent in the tumor. Using primary samples such as these, it is difficult to "reproduce" exact findings.
Randomization	Once each tumor biopsy was fragmented, a manual randomization approach was used to distribute the slices into separate wells of one plate. This process was used in order to capture as much heterogeneity in tissue across the two drug arms, as possible.
Blinding	Due to the limited number of scientists performing and analyzing the data from the experiments, we were not able to use a blinded approach.

Reporting for specific materials, systems and methods

We require information from authors about some types of materials, experimental systems and methods used in many studies. Here, indicate whether each material, system or method listed is relevant to your study. If you are not sure if a list item applies to your research, read the appropriate section before selecting a response.

Materials & experimental systems

n/a	Involved in the study	
☐	☒	Antibodies
☒	☐	Eukaryotic cell lines
☒	☐	Palaeontology and archaeology
☒	☐	Animals and other organisms
☒	☐	Human research participants
☒	☐	Clinical data
☒	☐	Dual use research of concern

Methods

n/a	Involved in the study	
☒	☐	ChIP-seq
☐	☒	Flow cytometry
☒	☐	MRI-based neuroimaging

Antibodies

Antibodies used	Nivolumab (Opdivo, BMS), isotype control IgG4 Ultra-LEAF purified human IgG4 isotype (Biolegend) anti-CD3-APC , anti-CD4-FITC, anti-CD8-PerCP-Cy5.5 anti- IFNgFITC/ anti-CD69 PE/ anti-CD8 PerCP-Cy™5.5/ anti-CD3 APC, 346048, BD Bioscience), T-reg Cocktail (anti-CD4 FITC/ anti-CD25 PE-Cy7/ anti-CD127 Alexa Fluor 647,560249, BD Bioscience) and anti-Foxp3 PE (560082, BD Bioscience). anti-CD45 AF700 (clone 560566), anti-CD4 Pe-Cy7 (557852), anti CD8-APC-H7(641400), anti-CD14 PE-Cy5.5 (562692), anti-Foxp3 PE (560082), all from BD Bioscience, anti-CD3 BV510 (317332, Biolegend). The live cells were gated using Live-Dead Blue fixable cell stain method (L23105, Thermo Fisher Scientific)
Validation	Validation is confirmed on the commercial site indicated by the above antibodies and sources

Flow Cytometry

Plots

Confirm that:

☒ The axis labels state the marker and fluorochrome used (e.g. CD4-FITC).

☒ The axis scales are clearly visible. Include numbers along axes only for bottom left plot of group (a 'group' is an analysis of identical markers).

☒ All plots are contour plots with outliers or pseudocolor plots.

☐ A numerical value for number of cells or percentage (with statistics) is provided.

Methodology

Sample preparation	Human tumor samples were dissociated using the Miltenyi Gentle MACS dissociator instrument and affliated tumor dissociation kit. Resulting single cell suspensions were stained and measured on the flow cytometer.
Instrument	BD Fortessa
Software	BD Diva software was used to collect the data and FlowJo was used to analyze the data.
Cell population abundance	*Describe the abundance of the relevant cell populations within post-sort fractions, providing details on the purity of the samples and how it was determined.*
Gating strategy	FSC-H/FSC-A plots were used to determine a single cell population, the resulting cells were subjected to FSC-A/APC-A to identify CD3+ lymphocyte population that were further gated into either CD8+ T cells (FSC-A/PerCP-Cy5.5) or CD4+ T cells (FSC-A/FITC). All gates were placed based on an original fluorescence minus one (FMO) experiment to determine appropriate gating.

☒ Tick this box to confirm that a figure exemplifying the gating strategy is provided in the Supplementary Information.
